# Regulation of the growth-to-ripening transition in tomato fruits by energy charge involving* SlATP-PRT* and *SlAPRT1*

**DOI:** 10.1186/s43897-026-00234-x

**Published:** 2026-07-02

**Authors:** Ye Liu, Peiwen Wu, Bowen Li, Zheng Bian, Guiqin Qu, Daqi Fu, Hongliang Zhu, Yunbo Luo, Weihao Wang, Benzhong Zhu

**Affiliations:** 1 The College of Food Science and Nutritional Engineering, China Agricultural University, Beijing, 100083 China; 2 The College of Food Science and Engineering, Shanxi Agricultural University, Shanxi, 030801 China

**Keywords:** Tomato fruit, Energy charge, Adenosine monophosphate, Histidine, Growth-to-ripening transition

## Abstract

The lifecycle of tomato fruit mainly comprises two distinct phases: the initial growth and development phase, followed by the ripening and senescence phase. However, the mechanism initially triggering the transition from an energy-intensive growth phase to the ripening phase remains unclear. First, we found that the fruit energy charge was significantly reduced before this transition, which might be caused by elevated levels of adenosine monophosphate (AMP) and histidine (His). Second, we constructed transgenic tomato plants for the key enzymes in the AMP salvage pathway (*SlAPRT1*) and His biosynthesis pathway (*SlATP-PRT*) via genome editing and overexpression. All transgenic plants caused a severe inhibition of fruit ripening, which was consistent with exogenous AMP treatment on wild-type plants, primarily due to the increased energy charge. Particularly, *Slatp-prt* mutant fruits could hardly produce ethylene or initiate ripening, but exogenous His treatment could restore their energy charge and ripening initiation. Finally, reducing the energy charge via exogenous His treatment also effectively accelerated the growth-to-ripening transition of wild-type fruits both on the plant and post-harvest. In conclusion, this study reveals that low energy charge and high His levels co-trigger the growth-to-ripening transition of tomato fruits, providing novel and valuable insights into the mechanisms underlying ripening initiation.

**Supplementary Information:**

The online version contains supplementary material available at 10.1186/s43897-026-00234-x.

## Core

SlATP-PRT and SlAPRT1 are key enzymes involved in the biosynthesis of AMP and His. The elevated levels of AMP and His are interrelated and crucial for reducing the energy charge during the later stage of tomato fruit growth. A low energy charge, combined with high His levels, serves as an essential prerequisite for the transition from growth to ripening.

## Gene & accession numbers

Sequence information of tomato genes in this study can be found in the Solanaceae Genomics Network (https://solgenomics.net/). The accession numbers are in Table S3.

The raw RNA-seq data and the mass spectrometry data have been deposited in the BIG Data Center Genome Sequence Archive (https://bigd.big.ac.cn/gsub/) under accession numbers CRA022104 and OMIX008650, respectively.

## Introduction

Fleshy fruits are the reproductive organs of flowering plants and are known for their delicious taste, vivid color, and sweet flavor. They are also rich in essential nutrients such as vitamins and minerals, making them an important part of a healthy diet for physical and mental well-being (Tang et al. 2020). Tomato fruit is considered a typical climacteric fruit and a model material for fruit ripening research (Giovannoni 2004; Giovannoni et al. 2017), whose life cycle mainly goes through two phases: the growth and development phase, and the ripening and senescence phase (Seymour et al. 2013). During the growth phase, tomato fruit undergoes cell division and expansion, increasing cell number and volume (Seymour et al. 2013). After the tomato fruit transitions from the growth phase to the ripening phase, it develops many edible characteristics such as color, aroma, texture, and nutrient composition through various physiological and biochemical processes such as pigment accumulation, aroma synthesis, and cell wall degradation (Chirinos et al. 2023). However, the physiological and molecular mechanisms governing the growth-to-ripening transition of tomato fruits remain unclear.

Generally, the initiation of tomato fruit ripening refers to the beginning of the ripening process, transitioning from the immature stage associated with fruit growth. The tomato fruit ripening process primarily includes the mature green (MG), breaker (Br), orange (Or), and red ripening (RR) stages, which are intricately regulated by various factors (Chen et al. 2020; Brumos 2021). Current research on tomato fruit ripening has mainly focused on the role and mechanism of hormones (primarily ethylene) (Fenn and Giovannoni 2021), environmental conditions (Adams et al. 2001; He et al. 2022), transcription factors (Jia et al. 2024), epigenetics (Li et al. 2022), and other aspects (Wang et al. 2023). First, ethylene is a crucial regulator of climacteric fruit ripening, particularly in tomatoes (Huang et al. 2022). The transition of tomato fruit from autoinhibitory ethylene production (system I) during the immature stage to autocatalytic ethylene production (system II) marks the beginning of ripening. In this process, ethylene promotes ripening only after the initiation of fruit ripening (Liu et al. 2015a; Giovannoni et al. 2017; Chirinos et al. 2023). However, the specific and initial mechanisms driving the transition of ethylene synthesis from system I to system II have yet to be clarified. Second, environmental conditions, such as temperature and blue light, also play significant roles in tomato fruit ripening. Elevated temperature (Adams et al. 2001) and supplemental blue light (He et al. 2022) both promote the initiation of tomato fruit ripening; however, the precise physiological and molecular mechanisms implicated remain elusive. Third, many transcription factors play an important role in regulating the initiation of tomato fruit ripening (Quinet et al. 2019; Chen et al. 2020). Among them, two key regulators, SlRIN and SlNOR, have been identified as crucial factors in tomato fruit ripening because their functional deficiencies lead to delayed ripening and inability to turn red (Ito et al. 2017; Li et al. 2018; Gao et al. 2020). Knocking out or silencing other important transcription factor-coding genes, such as *SlCNR* (Gao et al. 2019), *SlTAGL1* (Vrebalov et al. 2009), *SlNOR-like1* (Gao et al. 2018), and *SlNAM1* (Gao et al. 2021), has also been shown to delay the initiation of fruit ripening. In contrast, the downregulation of *SlAP2a* or *SlMADS1* through RNA interference was associated with the early initiation of fruit ripening (Chung et al. 2010; Dong et al. 2013). Despite these findings, the precise mechanisms by which these factors influence the transition of fruit from growth to ripening remain ambiguous. Finally, recent studies on epigenetic factors have emphasized the significance of DNA methylation and histone modification in regulating the initiation of fruit ripening (Liu et al. 2015b; Lü et al. 2018; Ding et al. 2022). Particularly, mutants of the *SlDML2* gene displayed considerable delays in the onset of tomato fruit ripening (Liu et al. 2015b; Lang et al. 2017). Nevertheless, the molecular mechanisms responsible for initiating fruit ripening have not been elucidated.

In addition to these factors, the energy status exerted a significant influence on the growth and ripening of tomato fruit (Colombié et al. 2017; Zhou et al. 2022; Xiong et al. 2023). Changes in energy status are invariably accompanied by modifications in energy supply and demand, which affect various biological processes such as plant metabolism and growth (Baena-González et al. 2020). The energy supply and demand during the growing phase are higher than those during the ripening phase, which constitutes a key feature of the tomato fruit lifecycle (Colombié et al. 2017). Therefore, we focused on the role and potential mechanisms of this shift in energy status in inducing the growth-to-ripening transition of tomato fruits. In this study, a gradual decline in energy charge [(ATP + 1/2ADP)/(ATP + ADP + AMP)] was observed in wild-type tomato fruits from the growth process to the ripening process. This is primarily due to the elevated levels of AMP, which is accompanied by increased His content. The AMP salvage pathway and His biosynthesis pathway are primarily accountable for the elevated levels of AMP during the later stage of plant growth (Rees et al. 2009; Ashihara et al. 2018). APRT and ATP-PRT are the key enzymes in these respective pathways (Rees et al. 2009; Ashihara et al. 2018). Interestingly, we observed a significant inhibition of fruit ripening initiation in *Slatp-prt* mutants, OE-*SlATP-PRT*, and OE-*SlAPRT1* fruits, mainly attributed to an elevated energy charge during ripening transition. However, exogenous His treatment on tomato plants reduced fruit energy charge and promoted the initiation of fruit ripening in both wild-type and *Slatp-prt* mutants. Furthermore, the increased His content is essential for triggering the ripening transition of harvested immature tomato fruits. Our findings reveal that the decreased energy charge and the increased His content co-induce the ripening transition of tomato fruits.

## Results

### Changes in energy charge and His content during the ripening transition of tomato fruit

**Fig. 1:**
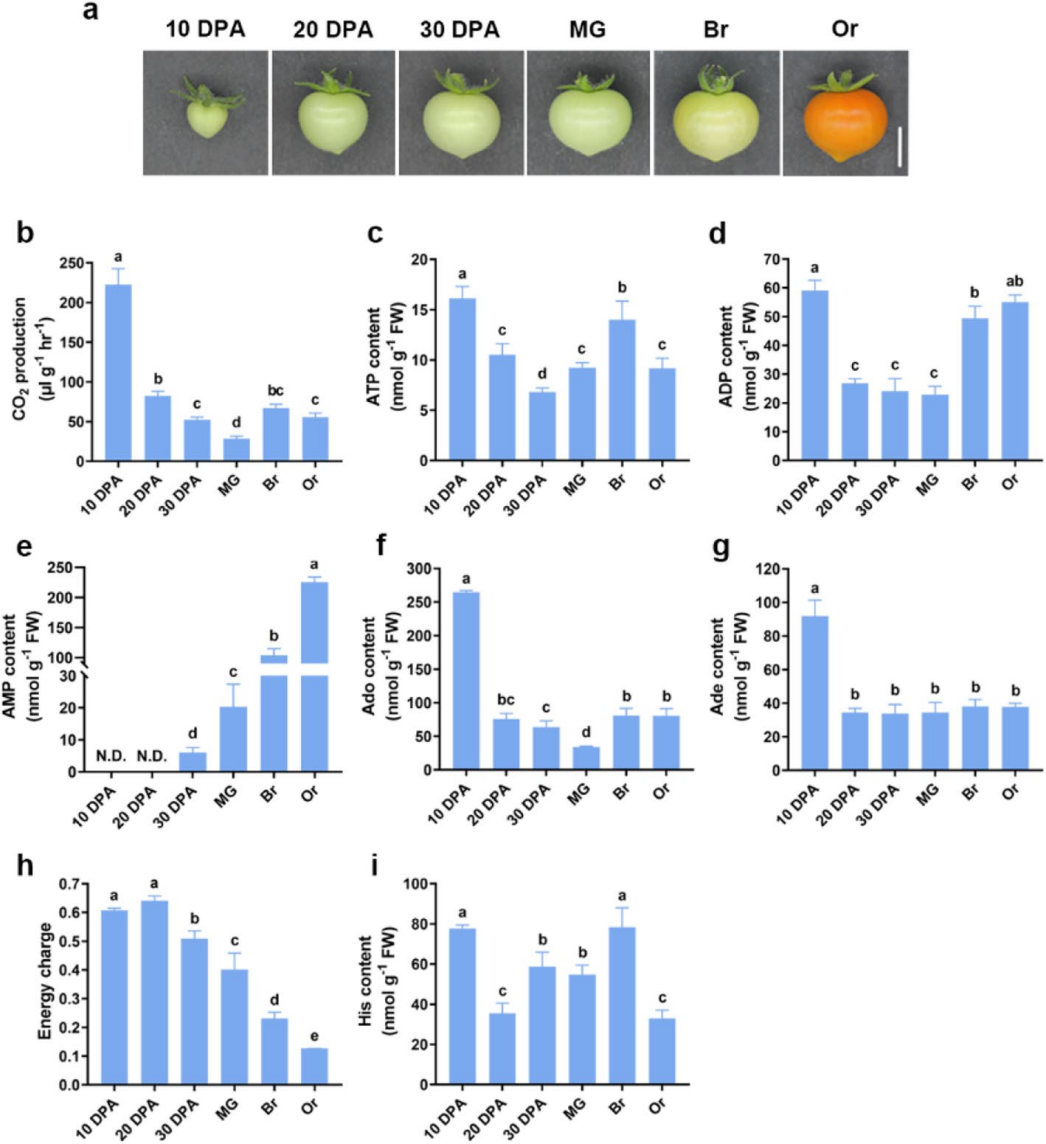
The cellular contents of energy charge-related metabolites and His in wild-type tomato fruits from growth to natural ripening. **a** The process from growth to natural ripening of wild-type tomato fruits. Bar = 1 cm, shown in the lower right corner. **b**-**i** The changes in CO_2_ production (**b**), ATP content (**c**), ADP content (**d**), AMP content (**e**), Ado content (**f**), Ade content (**g**), energy charge (**h**) and His content (**i**) of wild-type tomato fruits from growth to natural ripening. The error bars represent ± SD for three biological replicates. Different letters indicate significant differences as determined by multiple comparisons with Duncan's test (*P* < 0.05)

In this study, the cellular contents of energy charge-related metabolites such as ATP, ADP, AMP, adenosine (Ado), adenine (Ade), as well as CO_2_, and the energy charge of tomato fruits from the growth phase to the natural ripening phase were measured (Fig. 1a-h). During the early stage of fruit expansion (10–20 days post-anthesis, DPA), a significant reduction was observed in the levels of CO_2_, ATP, ADP, Ado, and Ade (Fig. 1b-d, f-g). Meanwhile, the AMP content remained undetectable (Fig. 1e), while the energy charge was maintained at a relatively high level (Fig. 1h). During the later stage of tomato fruit expansion (20–30 DPA), the ATP content declined to its lowest level, while the AMP content rose (Fig. 1c, e), leading to a significant decrease in energy charge (Fig. 1h). Nevertheless, the contents of ADP, Ado, and Ade remained stable throughout this period (Fig. 1d, f-g). During the fruit transition from growth to ripening (30 DPA-Br stage), there was a significant increase in ATP, ADP, and AMP levels, accompanied by a notable decrease in energy charge (Fig. 1c-e, h). Additionally, the Ado content decreased at the 30 DPA-MG stage but increased at the MG-Br stage (Fig. 1f), while the Ade content remained constant throughout this period (Fig. 1g). Furthermore, the CO_2_ production of the fruit reached its minimum at the MG stage, followed by a significant increase and a peak at the Br stage (Fig. 1b). These results imply that the contents of these energy charge-related metabolites undergo complex changes in the tomato fruit from growth to natural ripening. Among these metabolites, it is particularly noteworthy that both the energy charge and AMP content exhibit significant changes from 20 DPA to the Or stage. Specifically, the energy charge progressively decreases from 0.6 to 0.1, while the AMP content markedly increases from undetectable to 225.3 nmol g^−1^ FW, representing an order of magnitude increase (Fig. 1e, h). However, the contents of ATP and ADP fluctuated relatively slightly (Fig. 1c-d). Based on the formula for calculating energy charge, the alteration in energy charge in tomato fruits is predominantly driven by the changes in AMP content.

Furthermore, we observed that the content of free His in tomato fruits increased during the transition from growth to ripening (30 DPA-Br stage, Fig. 1i). This trend parallels the changes in ATP levels, which is consistent with the previously reported that ATP could promote His biosynthesis as a substrate of the rate-limiting enzyme ATP-PRT (Alifano et al. 1996). Additionally, His biosynthesis pathway in plants has been shown to be involved in the biosynthesis of AMP (Rees et al. 2009).

### Endogenous AMP and His biosynthesis are crucial for the transition of tomato fruit to ripening

The AMP salvage pathway and His biosynthesis pathway should be the major contributors to the increase of AMP levels in tomato fruits at the later stage of growth, and APRT and ATP-PRT are the key enzymes in these respective pathways (Rees et al. 2009; Ashihara et al. 2018). The amino acid sequences of APRT and ATP-PRT show high conservation among various species, including tomatoes, suggesting their evolutionary conservation and similar functional roles across different organisms (Fig. S1a-b). However, to the best of our knowledge, no research has been retrieved so far indicating that they are involved in the ripening and senescence processes of any species. In the tomato genome, *SlAPRT1* (*Solyc04g077970*) has a homologous gene identified as *Solyc08g079020*. However, the expression pattern of *Solyc08g079020* consistently declines during the fruit growth-to-ripening transition (Shinozaki et al. 2018). Additionally, the expression of *Solyc08g079020* (40.14 to 22.23 RPKM) was much lower than that of *SlAPRT1* (329.29 to 340.92 RPKM) during 30 DPA-to-Br stage (Shinozaki et al. 2018), indicating that *SlAPRT1* played a predominant role in this process. For *SlATP-PRT* (*Solyc01g103750*), it has no homologous counterpart in tomato. The expression of *SlAPRT1* and *SlATP-PRT* in the pericarp remained relatively stable throughout the growth-to-ripening transition (30 DPA-Br stage), and then decreased significantly in the later ripening stage, indicating their involvement in this transition process (Fig. S2a-f). Additionally, the expression level of *SlAPRT1* increased notably in locular tissue during this process (Fig. S2c). Recent reports have demonstrated that the expression of key ripening-related transcription factors, such as *SlRIN* and *SlNOR*, is initially induced in locular tissue before it occurs in the pericarp (Giovannoni et al. 2017; Shinozaki et al. 2018; Chirinos et al. 2023).

**Fig. 2:**
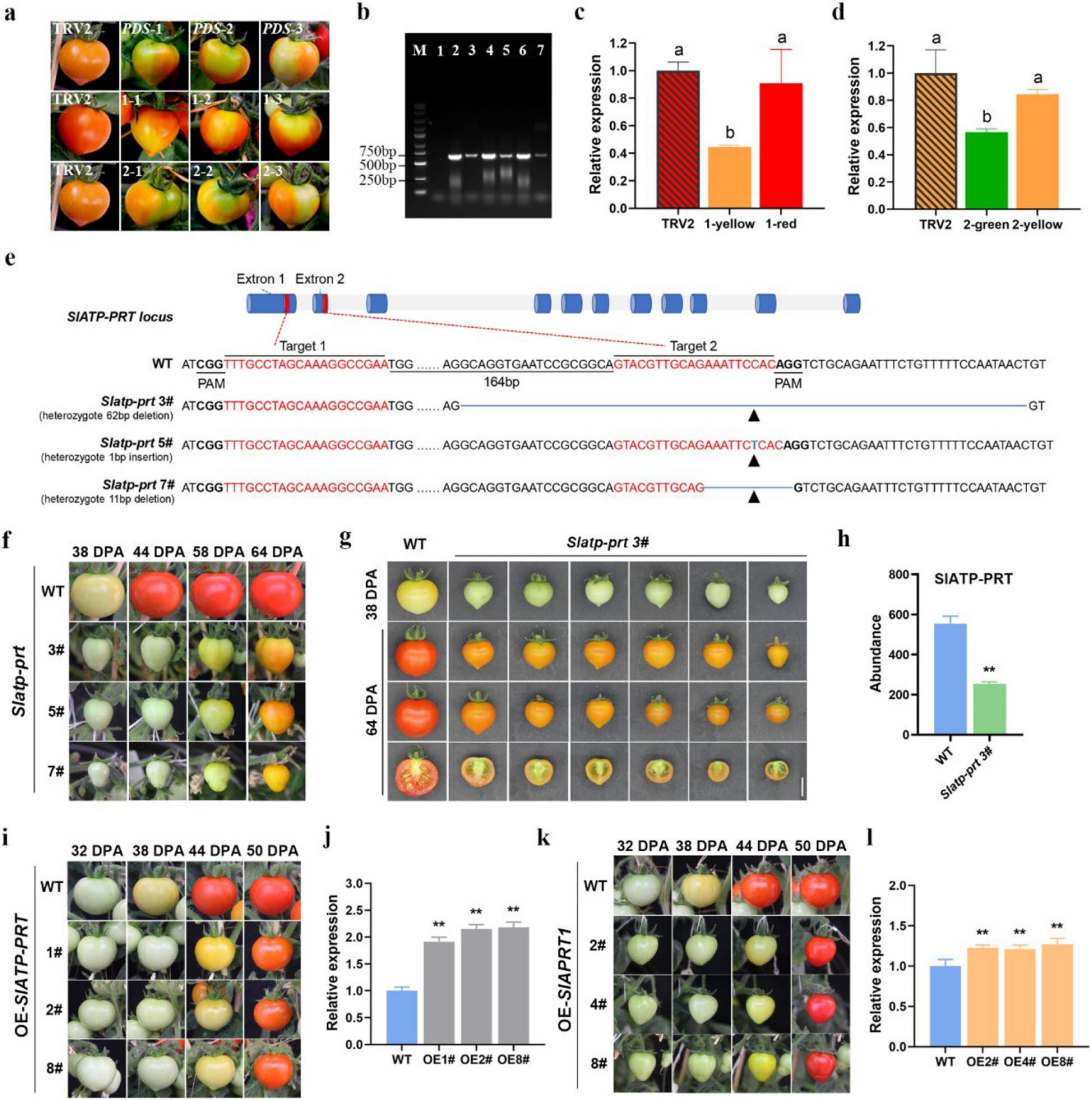
*SlATP-PRT* and *SlAPRT1* influence the growth-to-ripening transition of tomato fruit. **a** Phenotypes of silencing *PDS*, *SlATP-PRT* (1) or *SlAPRT1* (2) in wild-type fruits and negative control fruits (TRV2). **b** Detection of TRV virus in VIGS fruits. Lane 1, uninfected fruit; Lane 2&3, green part & yellow part of TRV2-*PDS* fruits; Lane 4&5, yellow part & red part of TRV2-*SlATP-PRT* fruits; Lane 6&7, green part & yellow part of TRV2-*SlAPRT1* fruits. **c** Relative expression of *SlATP-PRT* in the yellow and red parts of *SlATP-PRT-*silenced fruits and negative control fruits (TRV2). **d** Relative expression of *SlAPRT1* in the green and yellow parts of *SlAPRT1-*silenced fruits and negative control fruits (TRV2). *Slactin* is used as a reference in (**c**-**d**). The relative transcription levels of the control fruits were set to 1. The error bars represent ± SD for three biological replicates. Different letters indicate significant differences, as determined by multiple comparisons with Duncan's test (*P* < 0.05). **e** Genome editing site of the *SlATP-PRT* gene knockout mutants in the T1 generation. **f**-**g** Fruit ripening process and phenotypes of the T1 generation *SlATP-PRT* gene knockout mutants. Bar = 1 cm, shown in the lower right corner of (**g**). **h** Protein abundance of SlATP-PRT in the *Slatp-prt 3#* mutant and wild-type fruits (30 DPA) was analyzed by using the 4D-DIA proteomic technique. **i**-**j** Fruit ripening process (**i**) and relative expression (**j**) of *SlATP-PRT* in T1 generations OE- *SlATP-PRT*. **k**-**l** Fruit ripening process (**k**) and relative expression (**l**) of *SlAPRT1* in T1 generations OE-*SlAPRT1*. *Slactin* is used as a reference in (**j**) and (**l**). The relative transcription levels of the control fruits were set to 1. The error bars represent ± SD for three biological replicates. Compared with the wild-type fruits, ** indicates a statistically significant difference, determined by Dunnett's *t*-test (*P* < 0.01)

To regulate the energy charge and His content at the endogenous genetic level in tomatoes, we conducted instantaneous silencing, gene knockout, and gene overexpression experiments targeting the *SlAPRT1* and *SlATP-PRT*, respectively. First, the involvement of *SlATP-PRT* and *SlAPRT1* in tomato fruit ripening was successfully validated in Micro-Tom using virus-induced gene silencing (VIGS). VIGS targeting either *SlATP-PRT* or *SlAPRT1* resulted in delayed ripening of the fruits with an uneven color phenotype, and TRV sequences were detected in all of these fruits (Fig. 2a-d). Subsequently, to elucidate the specific roles of *SlATP-PRT* and *SlAPRT1* during tomato fruit ripening, their coding genes were knockout or overexpression in Micro-Tom. Notably, during the tissue culture, a substantial part of knockout and overexpressed seedlings did not survive (Fig. S3). Perhaps due to the indispensability of *SlATP-PRT* and *SlAPRT1* genes in tomatoes, knock-out homozygotes of either gene could not be obtained in the experimental process. As a result, we failed to get any *Slaprt1* heterozygotes mutants, but we have successfully generated three *Slatp-prt* heterozygotes mutants exhibiting functional deficiencies in SlATP-PRT (Fig. 2e). Additionally, we have established nine independent overexpression lines of *SlATP-PRT* and eight independent overexpression lines of *SlAPRT1*.

*Slatp-prt* 3#, 5#, and 7# all exhibited many severe deficiencies including fruit ripening, featuring a delayed Br stage of approximately 20 days and an inability to turn red (Fig. 2f-g), which is comparable to that observed in the well-known spontaneous mutant *nor* (Gao et al. 2020). The abundance of SlATP-PRT protein in the *Slatp-prt* 3# mutant fruits decreased significantly to 45.85% of that in wild-type fruits shortly before the transition to maturation (30 DPA) (Fig. 2h; Table S1). Previous studies have reported that mutations or gene silencing for most positive regulators of tomato fruit ripening result in a delay of the Br stage by approximately 2 to 10 days, such as SlNAC4 (Zhu et al. 2014), SlNAM1 (Gao et al. 2021), SlHY5 (Wang et al. 2021), CLASS-II KNOX (Keren-Keiserman et al 2022), MED25 (Deng et al. 2023). Nevertheless, the delay in the ripening transition observed in *Slatp-prt* mutants was more pronounced, suggesting that SlATP-PRT plays a more critical role in promoting the onset of tomato fruit ripening than these mostly reported positive regulators.

Interestingly, the overexpression of the *SlATP-PRT* gene can also impede the ripening transition of tomato fruit. The onset of fruit ripening in the OE-*SlATP-PRT* lines 1#, 2#, and 8# was markedly delayed, resulting in a delay of approximately 6 days at the Br stage (Fig. 2i). The expression levels of *SlATP-PRT* in these lines were approximately double those of in wild-type fruits (Fig. 2j). Similarly, the initiation of fruit ripening in OE-*SlAPRT1* lines 2#, 4#, and 8# was also significantly delayed, with a comparable delay of around 6 days at the Br stage (Fig. 2k). The expression levels of the corresponding genes in these lines increased to approximately 1.2 times that of wild-type fruits (Fig. 2l). Since ATP-PRT and APRT may competitively utilize the finite substrate (5-phosphoribosyl-1-pyrophosphate, PRPP), both are likely to influence AMP and His biosynthesis in plants (Fig. S1c-d, Koslowsky et al. 2008; Liu et al. 2023). Consequently, the observed alterations in the expression of *SlATP-PRT* or *SlAPRT1* do not directly reflect corresponding changes in AMP or His levels in the fruits. To elucidate the reasons for the delayed ripening observed in *Slatp-prt* mutant, OE-*SlATP-PRT*, and OE-*SlAPRT1* fruits, it was essential to evaluate the variations in AMP and His contents in these transgenic fruits and assess their impact on energy charge.

### *SlATP-PRT* and *SlAPRT1* primarily regulate the ripening transition of tomato fruits by modulating their energy charge

**Fig. 3:**
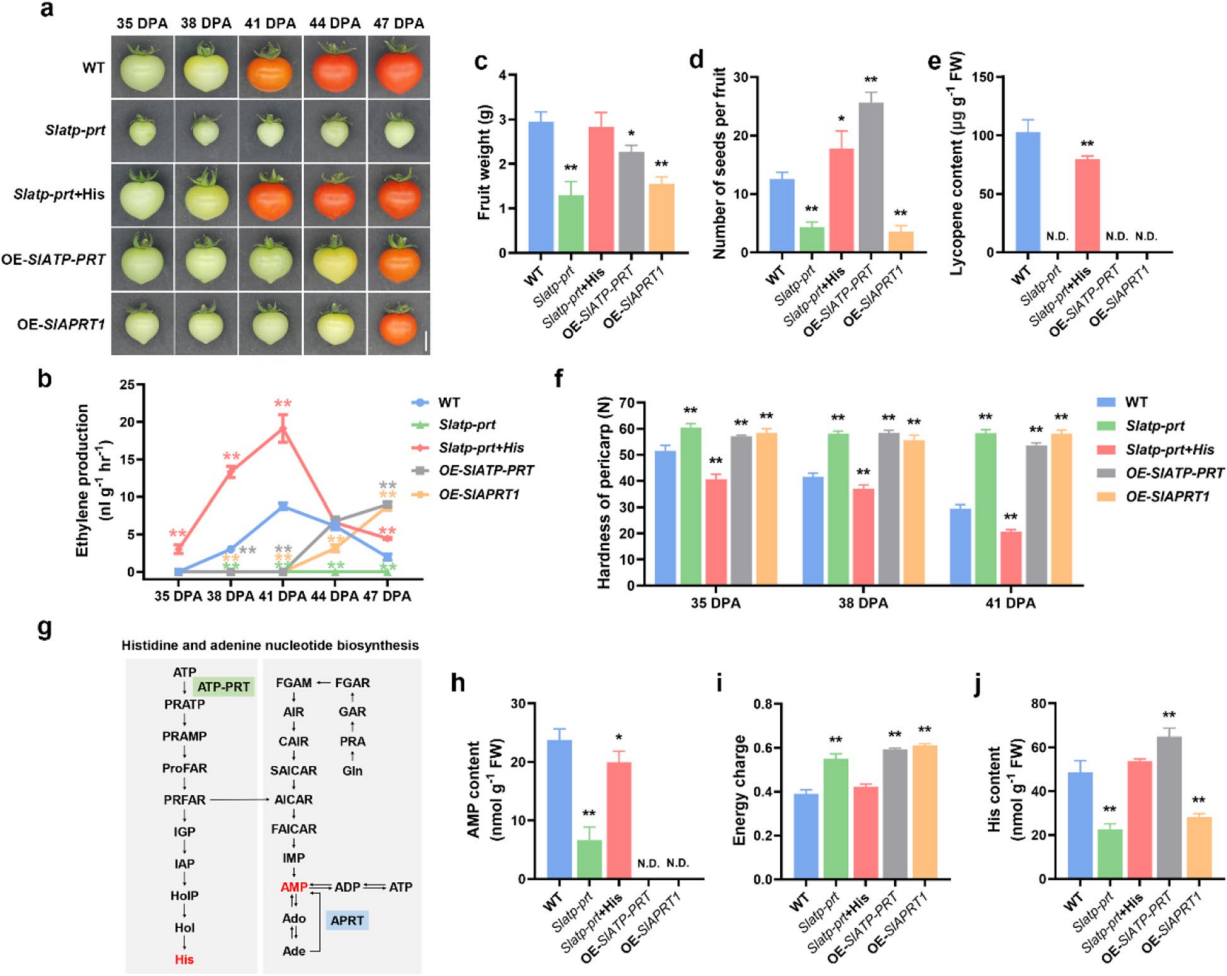
Endogenous AMP and His regulate the ripening transition of tomato fruit mainly by altering the energy charge. **a** Fruit ripening phenotypes of the T1 generation *Slatp-prt* mutant, the *Slatp-prt* mutant irrigated with 100 μmol L^−1^ His, the *SlATP-PRT* gene overexpression lines, and the *SlAPRT1* gene overexpression lines. Bar = 1 cm, shown in the lower right corner. **b** Ethylene production of the *Slatp-prt* mutant, the *Slatp-prt* mutant irrigated with 100 μmol L^−1^ His, OE-*SlATP-PRT*, and OE-*SlAPRT1* fruits from 35 to 47 DPA. **c**-**e** Fresh weight (**c**), the number of seeds per fruit (**d**), and lycopene content (**e**) in the *Slatp-prt* mutant, the *Slatp-prt* mutant irrigated with 100 μmol L^−1^ His, OE-*SlATP-PRT*, and OE-*SlAPRT1* fruits at 44 DPA. **f** The hardness of the *Slatp-prt* mutant, the *Slatp-prt* mutant irrigated with 100 μmol L^−1^ His, OE-*SlATP-PRT*, and OE-*SlAPRT1* fruits from 35 to 41 DPA. **g** The reported biosynthesis pathways of His (left) and AMP (right) involving ATP-PRT and APRT. The pathway above AMP is the de novo biosynthesis pathway for AMP, while the pathway below AMP is the salvage pathway for AMP. Abbreviations for metabolites are as follows: PRATP, 5-Phosphoribosyl ATP; PRAMP, 5-Phosphoribosyl AMP; ProFAR, Pro-phosphoribosyl formimino-5-aminoimidazole-4-carboxamide ribonucleotide; PRFAR, Phosphoribulosyl formimino-5-aminoimidazole-4-carboxamide ribonucleotide; IGP, Imidazoleglycerol phosphate; IAP, Imidazoleacetol phosphate; HolP, Histidinol phosphate; Hol, Histidinol; Gln, Glutamine; PRA, 5-Phosphoribosylamine; GAR, Glycinamide ribonucleotide; FGAR, Formylglycinamide ribonucleotide; FGAM, Formylglycinamidine ribonucleotide; AIR, 5-Aminoimidazole ribonucleotide; CAIR, 4-Carboxy aminoimidazole ribonucleotide; SAICAR, N-Succinyl-5-aminoimidazole-4-carboxamide ribonucleotide; AICAR, 5-Aminoimidazole-4-carboxamide ribonucleotide; FAICAR, 5-Formaminoimidazole-4-carboxamide ribonucleotide; IMP, Inosine monophosphate. **h**-**j** The AMP content (**h**), energy charge (**i**), and His content (**j**) in the *Slatp-prt* mutant, the *Slatp-prt* mutant irrigated with 100 μmol L^−1^ His, OE-*SlATP-PRT*, and OE-*SlAPRT1* fruits at 35 DPA. Error bars in (**b**-**f**) and (**h**-**j**) represent ± SD for three biological replicates. When compared with the wild-type tomato fruits, * (*P* < 0.05) and ** (*P* < 0.01) indicate a significant difference, as determined by Dunnett's *t*-test

Owing to their regulatory roles during the fruit ripening transition, *SlATP-PRT* and *SlAPRT1* exert significant influences on the development of ripening-associated physiological characteristics and quality attributes (Fig. 3a-f). Compared to wild-type tomato fruits, the His biosynthesis-deficient *Slatp-prt* mutant fruits showed almost complete inhibition of system II ethylene synthesis, fruit softening, and lycopene synthesis (Fig. 3b, e–f). Moreover, the weight of individual fruits and the number of seeds per fruit in *Slatp-prt* mutants were significantly lower than those in wild-type fruits (Fig. 3c-d). To ascertain whether the severe ripening defects observed in *Slatp-prt* mutants are solely attributable to the deficiency of His, we irrigated 100 μmol L^−1^ exogenous His to these mutants. Treatment with exogenous His significantly increased ethylene production and restored the ripening phenotypes of *Slatp-prt* mutant fruits (Fig. 3a-b). Furthermore, exogenous His treatment could significantly reduce the fruit hardness and increase lycopene content, single fruit weight, and seed number of single fruit in the *Slatp-prt* mutants (Fig. 3c-f). These findings proved that the genome-edited *Slatp-prt* mutant is a functionally deficient allele that completely impairs system II ethylene synthesis and fruit ripening initiation due to insufficient His biosynthesis. Moreover, the synthesis of system II ethylene was significantly delayed in both OE-*SlATP-PRT* and OE-*SlAPRT1* fruits compared to wild-type fruits (Fig. 3b). These transgenic fruits exhibited noticeable deficiencies in quality development (Fig. 3c-f). Overexpression of *SlATP-PRT* or *SlAPRT1* resulted in strong inhibition of fruit softening and lycopene synthesis, as well as a significant decrease in single fruit weight (Fig. 3c, e–f). In addition, the seed number of OE-*SlAPRT1* single fruit decreased significantly, while the seed number of OE-*SlATP-PRT* single fruit increased significantly compared to wild-type fruits (Fig. 3d). Overall, *SlATP-PRT* and *SlAPRT*, the key enzyme genes of AMP and His biosynthetic pathway play a crucial role in the growth-to-ripening transition of tomato fruits.

Because SlATP-PRT and SlAPRT1 participate in the biosynthesis of AMP and His, and thus affect the energy charge of tomato fruit (Fig. 3g, Liu et al. 2023). To further elucidate the mechanisms by which *SlATP-PRT* and *SlAPRT1* influence the initiation of fruit ripening, we measured the contents of His and AMP, and energy charge in *Slatp-prt* mutant, OE-*SlATP-PRT*, and OE-*SlAPRT1* fruits, as well as wild-type tomato fruits at 35 DPA (Fig. 3h-j). Compared with wild-type tomato fruits, the knockout of the *SlATP-PRT* gene resulted in decreased His and AMP contents, leading to an increased energy charge (Fig. 3h-j). Conversely, the overexpression of *SlATP-PRT* was associated with slightly elevated His content and reduced AMP content, also contributing to a higher energy charge (Fig. 3h-j). Additionally, the overexpression of *SlAPRT1* led to reductions in both His and AMP contents, thereby increasing the energy charge relative to wild-type tomato fruits (Fig. 3h-j). Unexpectedly, OE-*SlATP-PRT* and OE-*SlAPRT1* reduced AMP levels in 35 DPA fruits; nevertheless, this does not imply that the biosynthesis of AMP has not been enhanced. The observed reduction in AMP levels could be ascribed to its rapid conversion into other compounds since AMP is an essential metabolite for plant growth and development (Ashihara et al. 2018). Notably, all transgenic fruits exhibiting delayed ripening consistently showed a higher energy charge, indicating that an elevated energy charge is a key determinant of the delayed ripening phenotype.

Among these transgenic plants, the most significant delay in ripening was observed in the His-biosynthetic defective *Slatp-prt* mutants with a higher energy charge (0.6), which failed to initiate ripening within 47 DPA (Fig. 3a, i). However, exogenous His treatment down-regulated the energy charge (0.4) in *Slatp-prt* mutant fruits, thereby enabling the *Slatp-prt* mutants to transit to ripening as normally as wild-type fruits (Fig. 3a, i). These results suggest that adequate His is essential for reducing the energy charge in tomato fruits. Nevertheless, tomato fruits with high His content are not necessarily characterized by a low energy charge. For instance, although the His level in OE-*SlATP-PRT* fruits was marginally higher than that in wild-type fruits, their energy charge was also elevated; the 10 DPA wild-type fruit with a high His content also had a high energy charge.

### Exogenous energy charge-related metabolites treatment on tomato plants regulates fruit ripening transition

The energy charge is a complex parameter whose calculation formula incorporates the concentrations of ATP, ADP, and AMP, and it cannot be directly manipulated. Therefore, to confirm the effects of changes in energy charge on the initiation of fruit ripening, we employed treatment with exogenous energy charge-related metabolites to investigate their impact. Specifically, energy charge-related metabolites including ATP, inorganic phosphate (Pi), ADP, AMP, Ado, and Ade were individually applied to wild-type tomato plants, to modulate the energy charge of fruits during the immature stage. These metabolites can interconvert with each other, and these conversions are influenced by the levels of these metabolites within the cell (Moffatt and Ashihara 2002; Zrenner et al. 2006). Additionally, the early stage of fruit growth (10 and 15 DPA) was selected for the treatment of these metabolites in this study, because the rapid cell growth during this period facilitates their rapid conversion and utilization (Seymour et al. 2013), thus changing the energy charge of the fruit.

**Fig. 4:**
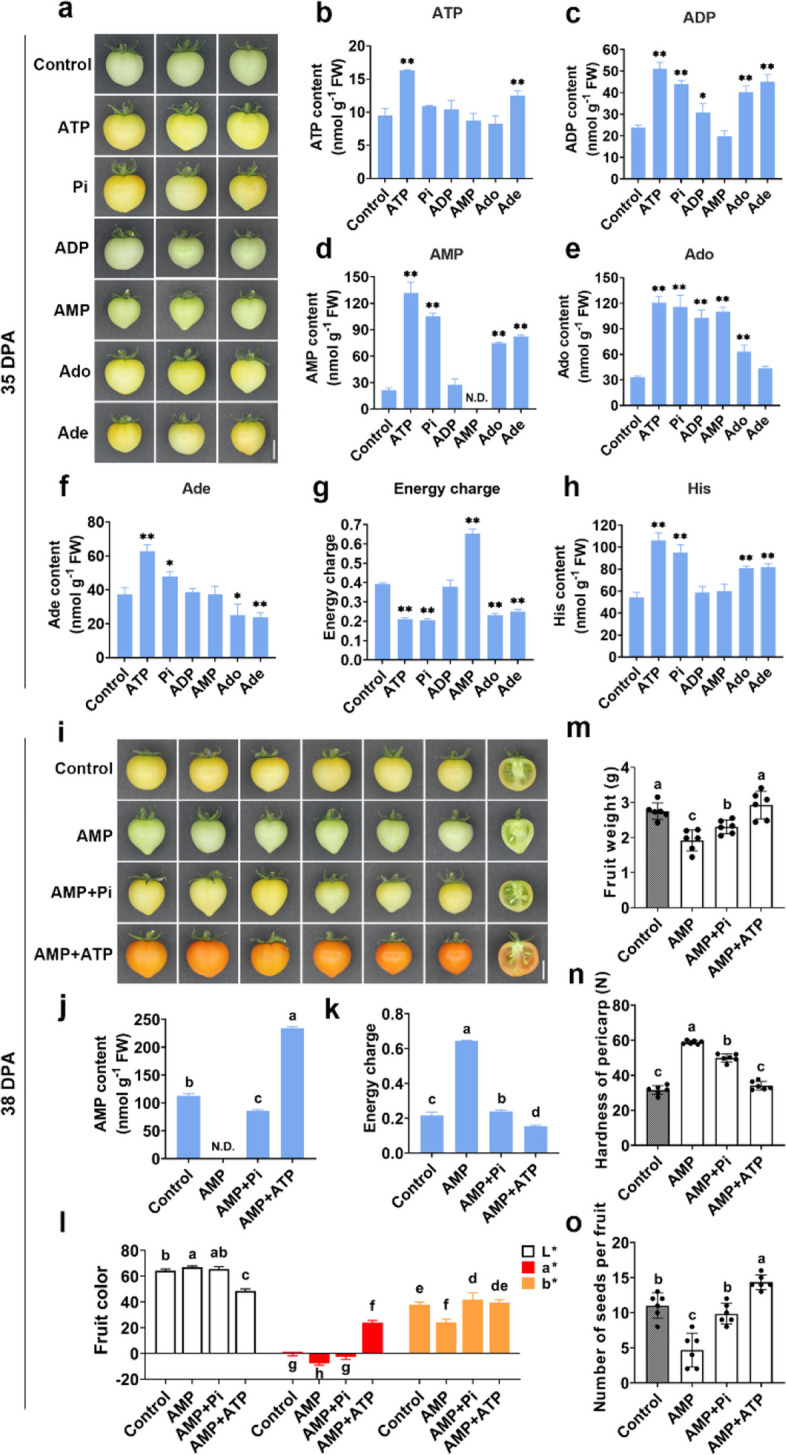
Exogenous energy charge-related metabolites regulate tomato fruit transition from growth to ripening. **a**-**h** The phenotype of fruit ripening (**a**), as well as ATP content (**b**), ADP content (**c**), AMP content (**d**), Ado content (**e**), Ade content (**f**), energy charge (**g**) and His content (**h**) of 35 DPA fruits after irrigating wild-type tomato plants with 100 μmol L^−1^ ATP, Pi, ADP, AMP, Ado or Ade at 10 and 15 DPA. The error bars represent ± SD for three biological replicates. Statistical analysis was performed by using Dunnett's *t*-test to compare with the negative control fruits; * (*P* < 0.05) and **(*P* < 0.01) indicated significant differences. **i**-**p** The ripening phenotype (**i**), as well as AMP content (**j**), energy charge (**k**), color (**l**), fresh weight (**m**), hardness (**n**), and seed number of single fruit (**o**) of 38 DPA fruits after treatment with 100 μmol L^−1^ AMP combined with equal molar quantities of Pi or ATP at 10 and 15 DPA. The error bars represent ± SD (*n* = 3 in **j**-**k**, and 6 in **l**-**o**). Different letters indicate significant differences as determined by multiple comparisons with Duncan's test (*P* < 0.05). Bar = 1 cm, shown in the lower right corner of (**a**) and (**i**)

When 100 μmol L^−1^ exogenous ATP, Pi, Ado, or Ade was applied to tomato plants, it led to an increased endogenous AMP content in the tomato fruits at 35 DPA (Fig. 4d), causing the energy charge to decrease to approximately 0.2 and accelerating the ripening transition of tomato fruits by approximately 3 days. (Fig. 4a, g). In contrast, exogenous ADP treatment had no significant effects on the energy charge of the fruits at 35 DPA (Fig. 4a, g). Interestingly, similar to those observed in OE-*SlATP-PRT* and OE-*SlAPRT1* fruits (Fig. 3a, h), exogenous AMP treatment also caused an abnormal decrease in endogenous AMP content in fruits at 35 DPA thereby increasing the cellular energy charge (Fig. 4d, g). Although the metabolic fate of AMP remains uncertain, these findings clearly indicate that a significant reduction in endogenous AMP levels is directly associated with an elevated energy charge. Furthermore, supplementing AMP-treated plants with equivalent molar amounts of Pi or ATP at 10 and 15 DPA effectively reduced the energy charge in AMP-treated fruits and thus compensated for the delayed ripening phenotype (Fig. 4i-k). Additionally, ATP supplementation significantly promoted color transformation, softening, and seed production (Fig. 4i, l-o). Therefore, the elevation in energy charge is the primary factor responsible for delaying the ripening transition in AMP-treated fruits. Based on the aforementioned findings, the decrease in energy charge in tomato fruits is an essential precondition for the growth-to-ripening transition.

### Exogenous His treatment on tomato plants promotes fruit energy charge reduction and ripening transition

**Fig. 5:**
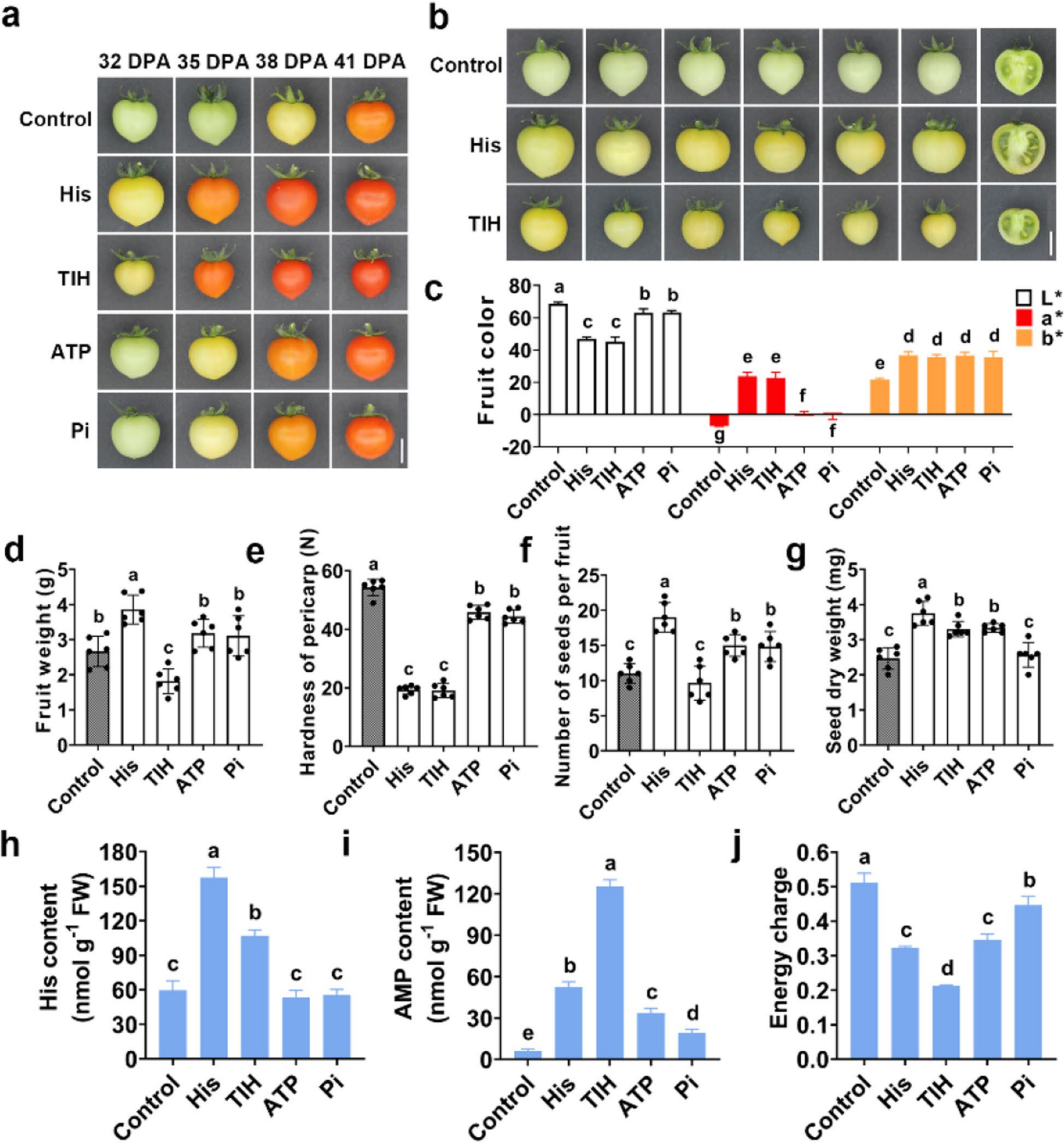
Exogenous His treatment of wild-type tomato plants facilitates fruit transition from growth to ripening. **a**-**j** The fruit ripening process (**a**), and 32 DPA fruit ripening phenotype (**b**), color (**c**), fresh weight (**d**), hardness (**e**), and 35 DPA single fruit seed number (**f**) and single seed dry weight (**g**), as well as His content (**h**), AMP content (**i**), energy charge (**j**) of 32 DPA fruits after irrigating wild-type tomato plants with 100 μmol L^−1^ His, TIH, ATP or Pi at 10 and 15 DPA. Bar = 1 cm, shown in the lower right corner of (**a**) and (**b**). The error bars indicate ± SD (*n* = 6 in **c**-**g**, and 3 in **h-j**). Different letters denote significant differences as determined by multiple comparisons with Duncan's test (*P* < 0.05)

Since the increase of fruit energy charge was found in *Slatp-prt* mutants with His deficiency (Fig. 3i-j), it was speculated that a high content of free His might promote the decrease of energy charge. However, the overexpression of *SlATP-PRT* could not substantially elevate the level of free His in fruits (Fig. 3j), so we employed exogenous supplementation to investigate its effects on energy charge and fruit ripening. 100 μmol L^−1^ His or ATP-PRT activator 3-(2-thienyl)-L-alanine (TIH) was utilized to irrigate wild-type tomato plants, respectively. Both His and TIH treatments significantly elevated His and AMP levels at 32 DPA, resulting in a notable decrease in fruit energy charge (Fig. 5h-j). Moreover, both exogenous His treatment and TIH treatment significantly advanced fruit ripening by approximately 6 days, and facilitated fruit color transformation and softening (Fig. 5a-c, e). Exogenous His treatment also promoted seed production in tomato fruits (Fig. 5f-g). Importantly, the treatment with TIH exhibited a more pronounced effect in reducing the energy charge of the fruit compared to treatments with equivalent concentrations of His, ATP, or Pi (Fig. 5j). Compared to ATP and Pi treatments, the His and TIH treatments led to earlier ripening initiation, color transformation, and softening of tomato fruits (Fig. 5a, c, e). These results confirmed that during the later stage of tomato fruit growth, the elevation of His level was highly effective in reducing the energy charge (Fig. 1h-i).

**Fig. 6:**
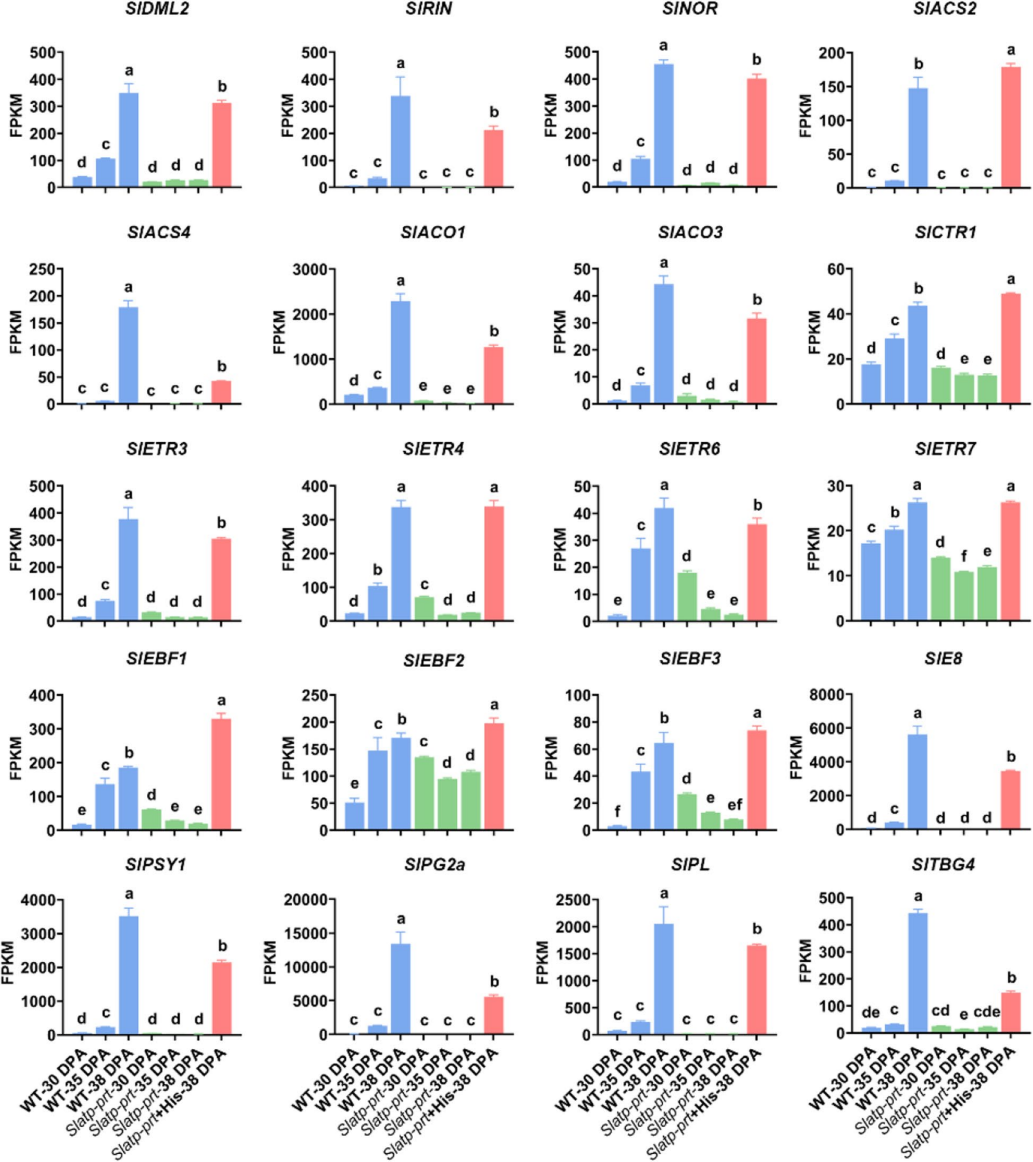
Endogenous His deficiency in the *Slatp-prt* mutant fruits repressed the expression of key ripening-related genes. Comparison of expression levels (FPKM) of genes associated with the initiation of ripening and quality formation in tomato fruits at various developmental stages of wild-type, *Slatp-prt* mutant, and *Slatp-prt* mutant irrigated with 100 μmol L^−1^ His. The accession numbers of these genes can be found in Table S3. The error bars represent ± SD for three biological replicates. Different letters indicate significant differences as determined by multiple comparisons with Duncan's test (*P* < 0.05)

Furthermore, RNA-seq analysis revealed that during the ripening transition, the differentially expressed genes in the His biosynthesis-deficient *Slatp-prt* mutant fruits and wild-type fruits were predominantly enriched in the plant hormone signal transduction pathway (Fig. S4). Among these genes, a substantial number of genes that were up-regulated by low energy charge in wild-type fruits were markedly repressed in *Slatp-prt* mutant fruits (Fig. 6). This was due to an increase in energy charge resulting from His deficiency (Fig. 3i-j). This repression covered key genes that promote or are involved in system II ethylene synthesis (*SlDML2*, *SlRIN*, *SlNOR*, *SlACS2*, *SlACS4*, *SlACO1*, *SlACO3*), signal transduction (*SlCTR1*, *SlETR3*, *SlETR4*, *SlETR6*, *SlETR7*, *SlEBF1*, *SlEBF2*, *SlEBF3*) and downstream response (*SlE8, SlPSY1, SlPG2a, SlPL, SlTBG4*) (Fig. 6, Fig. S5). Interestingly, the proteins encoded by these differentially expressed genes are almost all abundant in His residues. Among them, DNA demethylase SlDML2 displays the highest number of His residues per molecule (47), followed by ethylene receptors SlETR3 (20), SlETR4 (20), SlETR6 (21), and SlETR7 (21); the protein with the largest proportion of His is SlPL (17/403 = 4.22%), followed by SlNOR (13/355 = 3.66%) and SlRIN (9/280 = 3.21%). Significantly, exogenous His treatment markedly upregulated the energy charge and the expression of these differentially expressed genes in *Slatp-prt* mutant fruits, thereby effectively compensating for the ripening defects (Figs. 3a, i; 6). In summary, a low energy charge coupled with an elevated His level co-triggers the ripening transition in tomato fruits.

### His induces ripening transition in immature tomato fruits prior to system II ethylene production

It is widely recognized that harvested immature tomato fruits cannot initiate normal ripening due to the regulation by system Ⅰ ethylene, and no metabolites have been identified in tomato fruits that can effectively facilitate this process (Liu et al. 2015a; Giovannoni et al. 2017; Chirinos et al. 2023). Based on previous findings from this study, both ATP and His treatments significantly downregulated the energy charge in fruits at the later stage of growth (Fig. 5j). To investigate whether they play pivotal roles in the fruit growth-to-ripening transition, ATP and His were applied to tomato fruits at the late growth stage and their effects on the ripening initiation were evaluated.

**Fig. 7:**
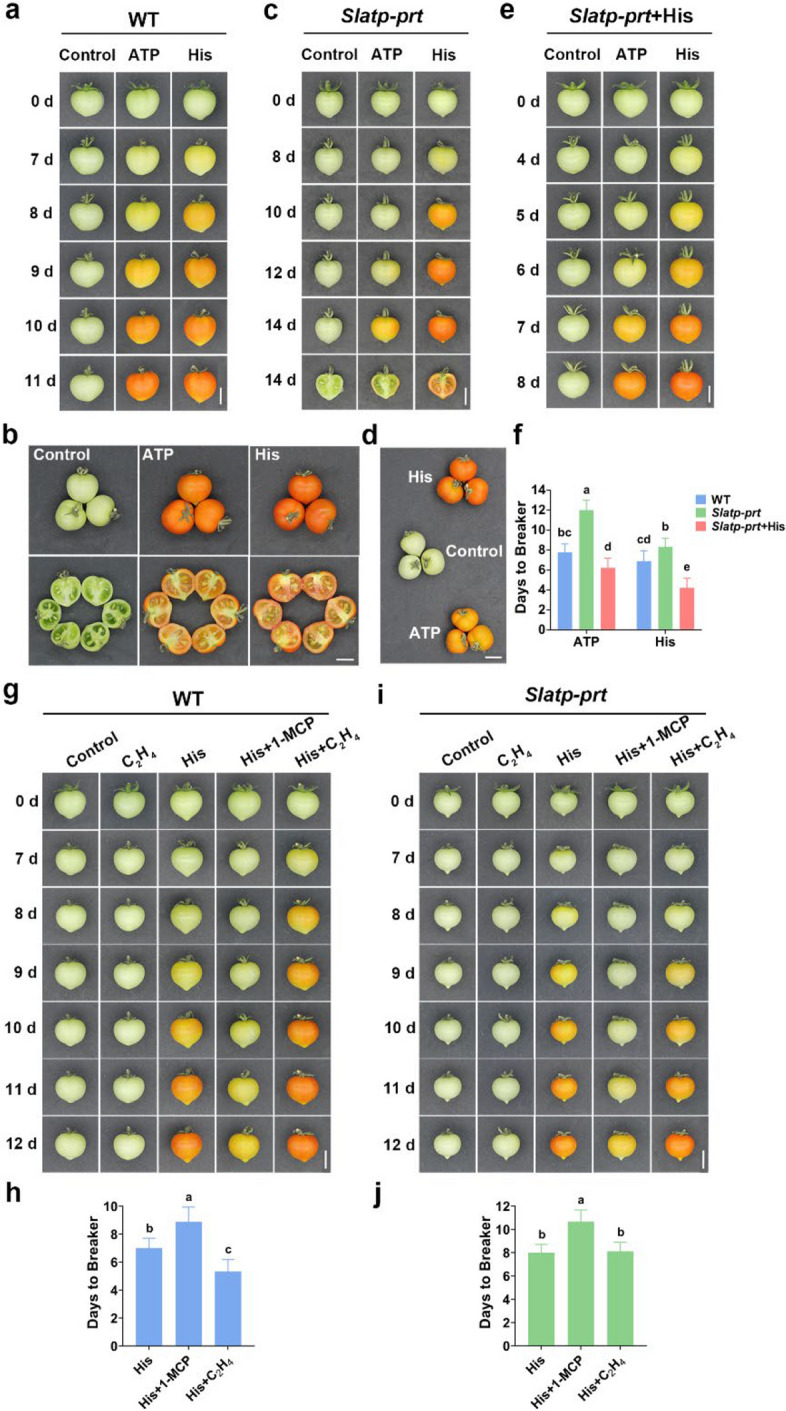
Exogenous ATP and His facilitate the transition of harvested immature tomato fruits to ripening*.* **a**-**b** The ripening process from 0 to 11 days (**a**) and the population diagram on day 11 (**b**) after the harvested wild-type fruits were treated with 100 μmol L^−1^ ATP or His injection. **c**-**d** The ripening process from 0 to 14 days (**c**) and the population diagram on day 20 (**d**) after the harvested *Slatp-prt* mutant fruits were treated with 100 μmol L^−1^ ATP or His injection. **e** Fruits harvested from *Slatp-prt* mutant plants treated with 10 μmol L^−1^ His were injected with either 100 μmol L^−1^ ATP or His, and their ripening process was observed for a period of 0–8 days. **f** The effect of 100 μmol L^−1^ ATP or His injection treatment on the Br stage of harvested wild-type and *Slatp-prt* mutant fruits with or without 10 μmol L^−1^ His irrigation. **g**-**h** The ripening process from 0 to 12 days (**g**) and the Br stage (**h**) after the harvested wild-type fruits were treated with ethylene fumigation, 100 μmol L^−1^ His injection combined with or without 50 ppm ethylene or 20 ppm 1-MCP fumigation. **i**-**j** The ripening process from 0 to 12 days (**i**) and the Br stage (**j**) after the harvested *Slatp-prt* mutant fruits were treated with ethylene fumigation, 100 μmol L^−1^ His injection combined with or without 50 ppm ethylene or 20 ppm 1-MCP fumigation. Wild-type and *Slatp-prt* mutant fruits were harvested at 26 DPA. Bar = 1 cm, shown in the lower right corner of (**a**-**e**), (**g**), (**i**). The error bars in (**f**), (**h**), and (**j**) represent ± SD for three biological replicates, and different letters indicate significant differences as determined by multiple comparisons with Duncan's test (*P* < 0.05)

As illustrated in Fig. 7a, harvested tomato fruits at the immature stage (26 DPA) fail to initiate the normal ripening process. Nevertheless, both ATP and His treatments were found to facilitate the onset of ripening of harvested immature wild-type fruits, ultimately leading to complete red coloration, and the effects of ATP and His treatments were comparable (Fig. 7a-b, f). Notably, the impact of ATP on fruit ripening in the His-deficient mutant *Slatp-prt* was significantly attenuated, resulting in a later Br stage compared to ATP-treated wild-type fruits and failure to turn red (Fig. 7c-d, f). Pre-watering *Slatp-prt* mutant plants with a low concentration (10 μmol L^−1^) of His enhanced the promoting effect of ATP on harvested immature *Slatp-prt* fruits to initiate ripening, leading to a faster color transition (Fig. 7c, e, f). Hence, our findings suggest that His plays a vital role in the ripening transition of tomato fruits induced by ATP treatment.

To further confirm whether His induces ripening transition in immature tomato fruits prior to system II ethylene production, the following investigations were carried out. Firstly, it was verified that exogenous ethylene treatment could not induce ripening in harvested 26 DPA tomato fruits, including wild-type and *Slatp-prt* mutant (Fig. 7g-j). In contrast, His treatment effectively facilitated the ripening initiation of these fruits, making them turn completely red (Fig. 7g-j). Moreover, the ethylene receptor inhibitor 1-MCP partially suppressed the His-induced initiation of fruit ripening, indicating that His acts partly through the ethylene-dependent pathway (Fig. 7g-j). Secondly, in spontaneous ripening-deficient mutants such as *rin*, *nor*, and *nr*, His treatment could advance the turning stage of harvested immature fruits, demonstrating its ability to promote the ripening transition via ethylene-independent pathways (Fig. S6). Nevertheless, due to the functional deficiencies of key ripening-related factors in these mutants (*rin*, *nor*, and *nr*), the fruits could not fully ripen to red (Ito et al. 2017; Li et al. 2018; Gao et al. 2020; Huang et al. 2022). Thirdly, in wild-type tomato fruits, the notable increase in His content (20–30 DPA) occurred before the significant up-regulation of transcription factors *SlRIN* and *SlNOR*, as well as *SlACS2* and *SlACS4* related to system II ethylene synthesis (Figs. 1i; 6; Fig. S7). Collectively, these findings imply that His-induced tomato fruit ripening transition is partly via the ethylene-dependent pathway and His acts prior to SlRIN, SlNOR, and system II ethylene.

## Discussion

Energy homeostasis is crucial for the growth and development of all organisms, necessitating a balance between energy demand and supply (Carling et al. 2011). The period of fruit growth is highly energy-consuming, yet the energy supply gradually diminishes as the fruit develops (Colombié et al. 2017). This deficiency in energy gives rise to a progressive decrease in ATP content throughout the growth process of fruits, ultimately resulting in a reduced energy state at the end of growth (Fig. 1c, h). Subsequently, the fruit enters a ripening stage marked by relatively lower energy demands (Colombié et al. 2017). During the ripening process, the patterns of energy supply differ between climacteric and non-climacteric fruits; climacteric fruits exhibit a respiratory peak that is absent in non-climacteric fruits (Klee and Giovannoni 2011; Gapper et al. 2013; Hewitt and Dhingra 2020). Nevertheless, both respiratory climacteric fruits (e.g., tomato) and non-respiratory climacteric fruits (e.g., litchi) exhibit an increase in ATP content due to excess energy supply during the ripening stage (Colombié et al. 2017; Zhou et al. 2022). It is evident that substantial alterations in energy supply and demand are involved in the growth-to-ripening transition of fruits, directly affecting the energy state of the fruits.

In this study, it was observed that the cellular energy charge decreased at the later stage of tomato fruit expansion (20–30 DPA), due to the lowest ATP content and a slight increase in AMP level (Fig. 1c, e, h). This low-energy state facilitates the upregulation of the expression of numerous ripening-related genes, including key regulatory genes such as *SlDML2*, *SlRIN*, and *SlNOR*, thereby promoting the ripening transition (Fig. 6). Such a low-energy state has been reported to be primarily sensed by AMP-activated protein kinase (AMPK), a highly evolutionarily conserved regulator of energy homeostasis in eukaryotes that exhibits high sensitivity to minor increases in AMP levels (Carling et al. 2011). In plants, the homolog of AMPK is SNF1-related kinase 1 (SnRK1), which is also activated by phosphorylation under a low-energy state (Crepin and Rolland 2019; Baena-González et al. 2020; Peixoto and Baena-González 2022). Upon activation, SnRK1 orchestrates extensive transcriptional reprogramming by phosphorylating downstream regulatory factors, including transcription factors (such as bZIP63, WRKYs, and NACs) and epigenetic factors (like H3K27me3 demethylase JMJ705) (Crepin and Rolland 2019; Liu et al. 2023). Notably, in tomato fruits, SnRK1 has been demonstrated to play a critical regulatory role during the transition to ripening under a low-energy state. Overexpression of apple *SnRK1* was found to accelerate the ripening initiation of transgenic tomato fruits by approximately 10 days (Li et al. 2010; Wang et al. 2012). Conversely, silencing of *SlSnRK1 (Solyc02g067030)* via VIGS led to delayed or complete inhibition of tomato fruit ripening, along with downregulation of numerous ripening-related genes, including *SlRIN*, *SlNOR*, *SlETR3*, and *SlCNR* (Lai et al. 2020). Moreover, SnRK1 directly interacts with SlCNR and phosphorylates it, facilitating its nuclear translocation (Lai et al. 2020). Collectively, these findings indicate that SnRK1 likely plays a central role in mediating transcriptional reprogramming in response to the low-energy state occurring during the later stage of tomato fruit growth.

The increased ATP content during the growth-to-ripening transition of tomato fruit (30 DPA-Br stage) serves as the booster for ripening initiation (Fig. 1c). This is because ATP is the foundation for all other nucleotide biosynthesis and is also a crucial substrate for the synthesis of numerous ripening-related factors, such as ethylene and His (Liu et al. 2023). Moreover, the exogenous ATP irrigation of tomato plants at 10 and 15 DPA increased the content of endogenous AMP to lower the energy charge at the later stage of tomato fruit growth, thus advancing the ripening transition (Fig. 4a, d, g). In addition, the injection of exogenous ATP into harvested immature tomato fruits (26 DPA) has been demonstrated to accelerate the ripening initiation (Fig. 7a). This finding contrasts with the effects observed when exogenous ATP treatments are applied to fruits that are already in the process of ripening. Previous studies have established a close association between decreased ATP levels and fruit ripening as well as senescence (Shan et al. 2023). Furthermore, exogenous ATP treatment has been shown to delay post-harvest ripening and senescence processes in ripening fruits such as apple (Shu et al. 2022), litchi (Zhou et al. 2022), and longan (Li et al. 2020). It is evident that exogenous ATP treatment yields varying effects on fruits depending on their developmental stages, likely due to fluctuations in endogenous ATP levels within the fruits. Specifically, the endogenous ATP level increased significantly during the growth-to-ripening transition of tomato fruits (30 DPA-Br stage) (Fig. 1c), and this elevated ATP level facilitated the initiation of fruit ripening (Figs. 4a; 7a). Nevertheless, after the ripening initiation of tomato fruits, the endogenous ATP level declined significantly during the subsequent ripening process (Br-Or stage) (Fig. 1c).

Furthermore, exogenous His could enhance the effect of ATP on the ripening initiation of harvested immature tomato fruits (Fig. 7a-f). Numerous studies have demonstrated that immature tomato fruits fail to ripen normally post-harvest and cannot be induced by ethylene, as ethylene synthesis has not transitioned from system Ⅰ to system II (Liu et al. 2015a; Giovannoni et al. 2017; Chirinos et al. 2023). Our findings demonstrate that His treatment reduces energy charge, thereby facilitating the growth-to-ripening transition of tomato fruits (Figs. 3a, i; 5b, j), although the underlying mechanism remains to be fully elucidated. Notably, this effect occurs prior to SlRIN, SlNOR, and system II ethylene involvement. This is supported by the observation that exogenous His treatment can induce ripening initiation in harvested immature fruits of spontaneous mutants (*rin*, *nor*, *nr*) as well as wild-type tomato fruits (Fig. 7g-h; Fig. S6). Furthermore, during natural fruit ripening, the up-regulation of related genes (*SlRIN*, *SlNOR*, *SlACS2,* and *SlACS4*) occurs subsequent to a significant increase in His content (Figs. 1i; 6; Fig. S7). In addition, the His-deficient *Slatp-prt* mutant fruits showed significant repression of gene expression for numerous ripening-associated proteins rich in His residues due to elevated energy charge at the MG stage. These proteins include those involved in DNA demethylation (e.g. SlDML2), transcription factors (e.g. SlRIN, SlNOR), system II ethylene synthesis, and signal transduction processes (e.g. SlETR3) (Fig. 6). Previous research has shown that the protein abundance of SlETR3 increases substantially during the MG and Br stages, while its transcript level remains relatively stable, indicating that translational efficiency or protein synthesis is the primary limiting factor for SlETR3 accumulation during this period (Mata et al. 2018). His, an essential amino acid vital for plant growth and development, is one of the least abundant amino acid residues in proteins, making up only 2.42% of total protein residues in tomatoes (Rees et al. 2009). However, it is present in relatively high proportions in the key ripening-related proteins mentioned above. As a result, the limited pool of free His may constrain the synthesis of these critical ripening-related proteins. Thus, the accumulation of His in tomato fruits may facilitate the ripening transition by enabling the efficient synthesis of these essential proteins.

**Fig. 8:**
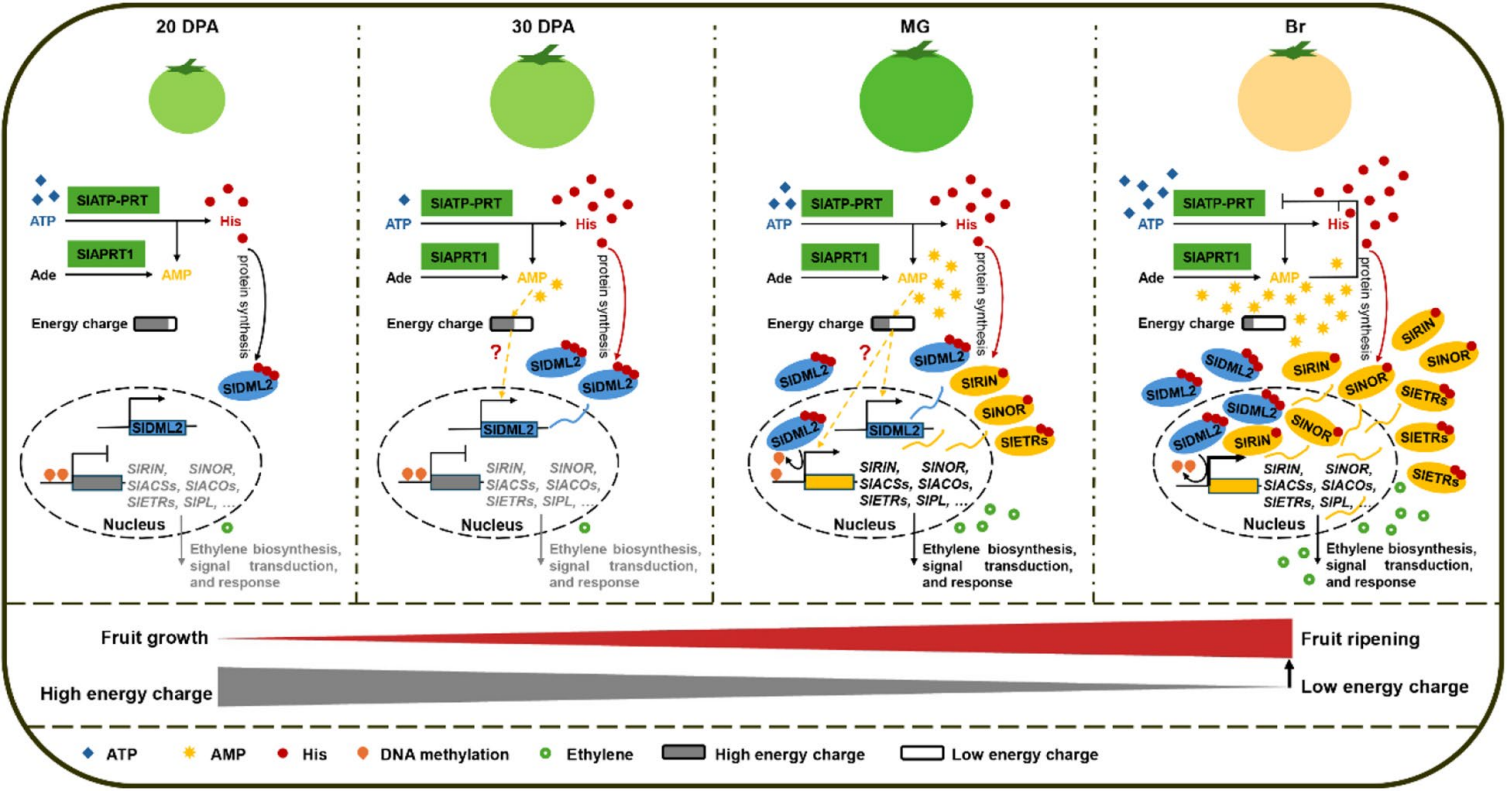
Hypothetical mechanisms by which the decreased energy charge induces the growth-to-ripening transition of tomato fruit. The elevation of AMP and His levels resulted in a reduction in fruit energy charge [(ATP + 1/2ADP)/(ATP + ADP + AMP)], thereby up-regulating the transcriptional expression of genes associated with DNA demethylation, transcription factors, as well as system II ethylene biosynthesis, signal transduction, and response. Concurrently, the increased His participated in the synthesis of these proteins, leading to a considerable system II ethylene synthesis and initiating fruit ripening

In summary, this study highlights the crucial role of reduced energy charge and elevated level of free His in inducing the growth-to-ripening transition of tomato fruits, along with proposed mechanistic hypotheses (Fig. 8). The increase in AMP and His levels led to a reduction in energy charge during the later stage of tomato fruit growth. This decrease in energy charge induces extensive transcriptional reprogramming, including the up-regulation of gene expression associated with ripening initiation. At the onset of tomato fruit ripening, the increase in free His level promotes the synthesis of ripening-related proteins such as SlDML2, SlRIN, SlNOR, and SlETR3. Eventually, this process triggers the synthesis of system II ethylene and the ripening transition of tomato fruits.

## Materials and methods

### Plant materials and growth conditions

The tomato cultivar *Solanum lycopersicum* cv. MicroTom was utilized as the parental line for wild-type and the ripening-impaired mutants *nr*, *rin*, and *nor*. Wild-type seeds were purchased from the C.M. Rick Tomato Genetics Resource Center and bred in the laboratory. *Nr*, *rin*, and *nor* were all obtained from the laboratory of Professor Mingchun Liu (Sichuan University, China). The tomato was cultivated in a controlled greenhouse environment with a 16 h light/8 h darkness cycle at 23 ± 2 °C, accompanied by 60% relative humidity and weekly irrigation using a plant nutrient solution. Flowers were marked on the day of anthesis to assess the ripening stage of the fruit. Wild-type fruits were harvested at approximately 35 DPA for MG stage, 38 DPA for Br stage, 41 DPA for Or stage, and 44 DPA for RR stage. Fruits from *Slatp-prt* mutants, OE-*SlATP-PRT*, and OE-*SlAPRT1* overexpressed lines were harvested at the same DPA as those of wild-type fruits. Fruit samples were simultaneously harvested from six randomly selected individual plants. The samples were immediately frozen in liquid nitrogen and stored at −80 °C until use.

### Primers

All the primers designed and used in this study are listed in Table S2.

### VIGS experiment

According to the protocol previously described by Li et al. (2018), infecting solutions were prepared for VIGS targeting *SlATP-PRT*, *SlAPRT1*, and *PDS* genes, respectively. RT-qPCR was utilized to evaluate the relative expression of target genes in different colored fruit parts and negative control fruit. Gene expression levels were determined using the 2^−ΔΔCt^ analysis method, with samples normalized against *SlACTIN* (*Solyc11g005330*). Each experiment consisted of three distinct biological replicates.

### Analysis of gene expression patterns

The expression data for *SlATP-PRT* and *SlAPRT1* was obtained from the RNA-seq data of *S.lycopersicum* cv M82 Fruit (Shinozaki et al. 2018). The visualization of this data was conducted using the Tomato Expression Atlas database (http://tea.solgenomics.net/). Additionally, the pericarp of wild-type tomato fruits was collected at 10 DPA, 20 DPA, 30 DPA, MG stage, Br stage, Or stage, and RR stage. RNA was extracted and reverse transcription was carried out to evaluate the relative expression levels of the *SlATP-PRT* and *SlAPRT1* genes by RT-qPCR.

### Construction and identification of transgenic plants

The CRISPR/Cas9 series vector, modified by Yaoguang Liu's laboratory at South China Agricultural University, was utilized for *SlATP-PRT* or *SlAPRT1 *gene knockout following the method of Ma et al. (2015). To generate overexpressed plants, the coding region of *SlATP-PRT* or *SlAPRT1*, and HA tag sequences were amplified by PCR and inserted into the overexpression vector pCambia1302 controlled by the CaMV 2 × 35S promoter. The *Agrobacterium tumefaciens*-mediated genetic transformation was performed as previously reported (Wang et al. 2017). Tissue culture seedlings not infected by *A. tumefaciens* served as negative controls. The method of transgenic seedlings identification is described in Document S1.

### Quantitative proteomics

Protein quantification was conducted using the four-dimensional-data-independent acquisition (4D-DIA) technique to detect differences in the abundance of SlATP-PRT in wild-type and *Slatp-prt* mutant fruits. The fruit samples were harvested shortly before the initiation of ripening (30 DPA), and protein extraction was carried out using the method described by Wang et al. (2017). The proteomic analysis of 4D-DIA was conducted by Shanghai Majorbio Biopharm Technology Co., Ltd. (Shanghai, China).

### Determination of fruit fresh weight, hardness, color, and lycopene content

The weight of tomato fruit (44 DPA) was measured with an electronic balance (1/1000). The color and firmness of the pericarp of 35–41 DPA fruits were assayed as described previously (Li et al. 2018). The lycopene was extracted from the pericarp of 44 DPA fruits according to the previously described method (Huang et al. 2022). The lycopene content was measured using an UltiMate™ 3000 rapid separation liquid chromatography system, following the manufacturer's instructions. The identification of lycopene was based on retention time, and the lycopene content per gram of fresh fruit weight was calculated by using standard curves and expressed as μg g^−1^ FW.

### The assay of ethylene and carbon dioxide production

The ethylene production of tomato fruit was determined according to the method described by Wang et al. (2021). Additionally, the CO_2_ emission of tomato fruits was measured using a portable CO_2_ analyzer (Felix™, F-950) to reflect their respiratory intensity.

### Determination of ATP, ADP, AMP, Ado, Ade, and free His contents

In each experimental group, at least 9 tomato fruits of similar shape, size, and color were collected for metabolite determination. Each fruit (excluding the seed) was dissected into 4–6 small pieces along the columella, immediately frozen in liquid nitrogen, and then placed at −80 ℃ for further use. Before metabolite extraction, approximately 2.0 g samples were taken ground in liquid nitrogen, and thoroughly mixed. The content of ATP, ADP, AMP, Ado, and Ade was determined by high-performance liquid chromatography, while the free His content was determined by ultra-high-performance liquid chromatography/mass spectrometer. The procedures for extraction and determination are detailed in Document S1. Subsequently, the fruit energy charge was calculated using the formula [(ATP + 1/2 ADP)/(ATP + ADP + AMP)]. Three distinct biological replicates of each experimental group were included.

### Treatment of tomato plants and harvested fruits with energy charge-related metabolites

Wild-type tomato plants were respectively irrigated with solutions containing 100 μmol L^−1^ ATP, ADP, AMP, Ado, Ade, Pi, His, or the SlATP-PRT activator TIH (Pisco et al. 2017). Meanwhile, the negative control group was irrigated with a solution devoid of these metabolites. Each treatment group consisted of 12 tomato plants, and each plant received approximately 100 mL of the corresponding solution per irrigation event. The plants were irrigated once on the 10th and 15th days subsequent to the blooming of the first batch of flowers (typically 3–5 flowers at the top of each of 2–3 inflorescences on the main stem), ensuring that the soil in the pot (90*100 mm) was thoroughly moistened. Only the fruits from the first batch of blooming flowers were utilized for the subsequent research. Similar to wild-type plants, T1 generation *Slatp-prt* mutant plants were irrigated with either 10 μmol L^−1^ His or 100 μmol L^−1^ His, and the irrigation was repeated three times from flowering to early fruit growth.

Tomato fruits at the immature stage were placed in a plastic basket for 4 h after harvest. Approximately 200 μL 4-morpholineethanesulfonic acid (MES) buffer (10 mmol L^−1^ pH 5.5) containing 100 μmol L^−1^ ATP or His was injected into the upper columella of each fruit in the treatment group with a 1 mL sterile syringe. Fruits injected with MES buffer alone were used as negative controls. In addition, ethylene, and the combination of His and ethylene or 1-methylcyclopropene (1-MCP) for the treatment of immature tomato fruit is described in Document S1. There were three biological replicates per group, totaling 9 fruits, all of which shared similar shapes, sizes, and colors. The plastic basket was covered with plastic wrap for moisturization and placed in a constant light greenhouse (16 h light/day). The injection was repeated three days later, and then the ripening phenotype was observed and recorded.

### RNA sequencing

Wild-type tomato fruits at different developmental stages (30 DPA, 35 DPA, and 38 DPA) and *Slatp-prt* mutant fruits at the corresponding stages were collected for analysis. Additionally, 38 DPA fruits of *Slatp-prt* mutant plants treated with exogenous 100 μmol L^−1^ His were also included. Each experimental group comprised three distinct biological replicates, totaling at least 9 fruits. Total RNA was extracted from the entire fruit excluding the seed, as gene expression in various fruit tissues, including but not limited to the pericarp, is associated with ripening initiation (Chirinos et al. 2023). RNA-seq was conducted on the NovaSeq 6000 platform (Illumina) by Novogene Co., Ltd. (Beijing, China). The analysis of RNA-seq data was following the previously described method (Li et al. 2018). The expression levels of six differentially expressed genes (*SlRIN*, *SlNOR*, *SlACS2*, *SlACS4*, *SlDML2*, and *SlETR3*) were validated through RT-qPCR using the 2^−ΔΔCt^ analysis method, based on biological triplicates that were independent of the sequencing batch.

## Supplementary Information

Additional file 1: Figure S1. Amino acid sequence alignment of SlAPRT1 and SlATP-PRT and their catalytic reactions. Figure S2. Expression patterns of *SlAPRT1* and *SlATP-PRT* in tomato fruits from growth to ripening. Figure S3. Seedling phenotypes during tissue culture. Figure S4. KEGG pathway enrichment analysis of differentially expressed genes in *Slatp-prt* mutant fruits compared with wild-type fruits. Figure S5. The relative expression levels of key ripening-related genes in *Slatp-prt* mutant fruits compared with wild-type fruits. Figure S6. Exogenous ATP and His induced ripening transition in harvested immature spontaneous tomato mutants. Figure S7. Expression patterns of *SlRIN*, *SlNOR*, *SlACS2*, and *SlACS4* in tomato pericarp.

Additional file 2: Table S1. Protein abundances in wild-type and *Slatp-prt* mutant fruits at 30 DPA detected by the 4D-DIA proteome. Table S2. Primer sequences used in this study. Table S3. The accession numbers of tomato genes in this study.

Additional file 3: Document S1. Supplementary methods in this study.

## Data Availability

All data supporting the findings of this study are included in the manuscript and its online Supplementary Information. The raw RNA-seq data and the mass spectrometry data have been deposited in the BIG Data Center Genome Sequence Archive (https://bigd.big.ac.cn/gsub/) under accession numbers CRA022104 and OMIX008650, respectively.

## Abbreviations

AMP: Adenosine monophosphate
His: Histidine
MG: Mature green
Br: Breaker
Or: Orange
RR: Red ripening
ATP: Adenosine triphosphate
ADP: Adenosine diphosphate
Ado: Adenosine
Ade: Adenine
DPA: Days-post-anthesis
PRPP: 5-Phosphoribosyl-1-pyrophosphate
Pi: Inorganic phosphate
TIH: 3-(2-Thienyl)-L-alanine
ATP-PRT: ATP phosphoribosyl transferase
APRT: Adenine phosphoribosyl transferase
RPKM: Reads per kilobase per million mapped reads
VIGS: Virus-induced gene silencing
PDS: Phytoene desaturase
RT-qPCR: Quantitative real-time polymerase chain reaction
4D-DIA: Four-dimensional-data-independent acquisition
1-MCP: 1-Methylcyclopropene
AMPK: AMP-activated protein kinase
SnRK1: SNF1-related kinase 1
MES: 4-Morpholineethanesulfonic acid
FPKM: Fragments per kilobase million
KEGG: Kyoto encyclopedia of genes and genomes
