# Supplementary material for: Regulation of the growth-to-ripening transition in tomato fruits by energy charge involving SlATP-PRT and SlAPRT1
Source: Mol Hortic. 2026 Jul 2;6:48. doi: 10.1186/s43897-026-00234-x (PMC13326308; doi:10.1186/s43897-026-00234-x)
Supplement: Supplementary file 1 — Additional file 1: Figure S1. Amino acid sequence alignment of SlAPRT1 and SlATP-PRT and their catalytic reactions. Figure S2. Expression patterns of SlAPRT1 and SlATP-PRT in tomato fruits from growth to ripening. Figure S3. Seedling phenotypes during tissue culture. Figure S4. KEGG pathway enrichment analysis of differentially expressed genes in Slatp-prt mutant fruits compared with wild-type fruits. Figure S5. The relative expression levels of key ripening-related genes in Slatp-prt mutant fruits compared with wild-type fruits. Figure S6. Exogenous ATP and His induced ripening transition in harvested immature spontaneous tomato mutants. Figure S7. Expression patterns of SlRIN, SlNOR, SlACS2, and SlACS4 in tomato pericarp. [file 43897_2026_234_MOESM1_ESM.docx]

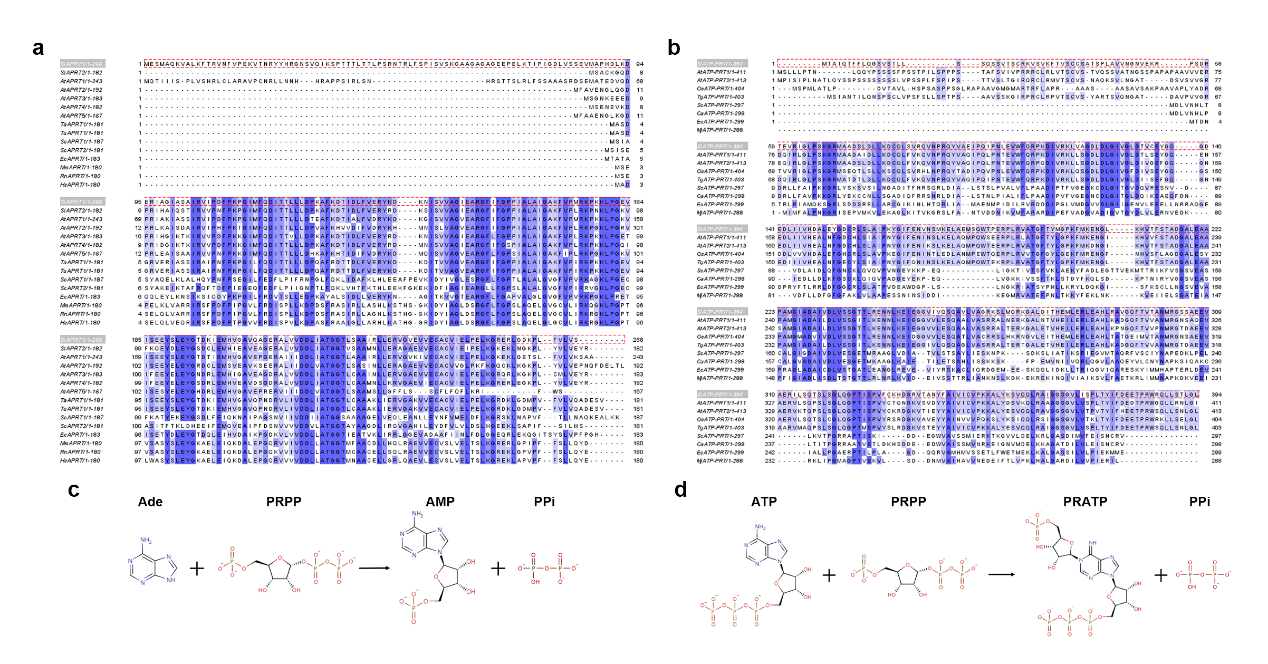


**Figure S1. Amino acid sequence alignment of SlAPRT1 and SlATP-PRT and their catalytic reactions.**

**a**, Amino acid sequence alignment of SlAPRT1 with its homologous proteins. *Arabidopsis thaliana*: AtAPRT1 (NP_564284.1), AtAPRT2 (NP_178122.1), AtAPRT3 (NP_193988.1), AtAPRT4 (NP_00131991.1), AtAPRT5 (NP_001329978.1); *Triticum aestivum*: TaAPRT1 (XP_044387050.1); *Triticum Urartu*: TuAPRT1 (XP_048548638.1); *Saccharomyces cerevisiae*: ScAPRT1 (NP_013690.1), ScAPRT2 (NP_010729.3); *Escherichia coli*: EcAPRT (AAN79066.1); *Mus musculus*: MmAPRT (NP_033828.2); *Rattus norvegicus*: RnAPRT (EDL92756.1); and *Homo sapiens*: HsAPRT (NP_000476.1).

**b**, Amino acid sequence alignment of SlATP-PRT with its homologous proteins, including *Arabidopsis thaliana*: AtATP-PRT1 (BAA89268.1), AtATP-PRT2 (BAA89269.1); *Oryza sativa*: OsATP-PRT (XP_015631722.1); *Thlaspi goesingense*: TgATP-PRT (AAB88880.1); *Saccharomyces cerevisiae*: ScATP-PRT (NP_010975.3); *Candida albicans*: CaATP-PRT (CAA58751.1); *Escherichia coli*: EcATP-PRT (NP_416523.1); *Methanococcus jannaschii*: MjATP-PRT (WP_010870716).

**c**-**d**, The chemical reaction catalyzed by SlAPRT1 (**c**) and SlATP-PRT (**d**).


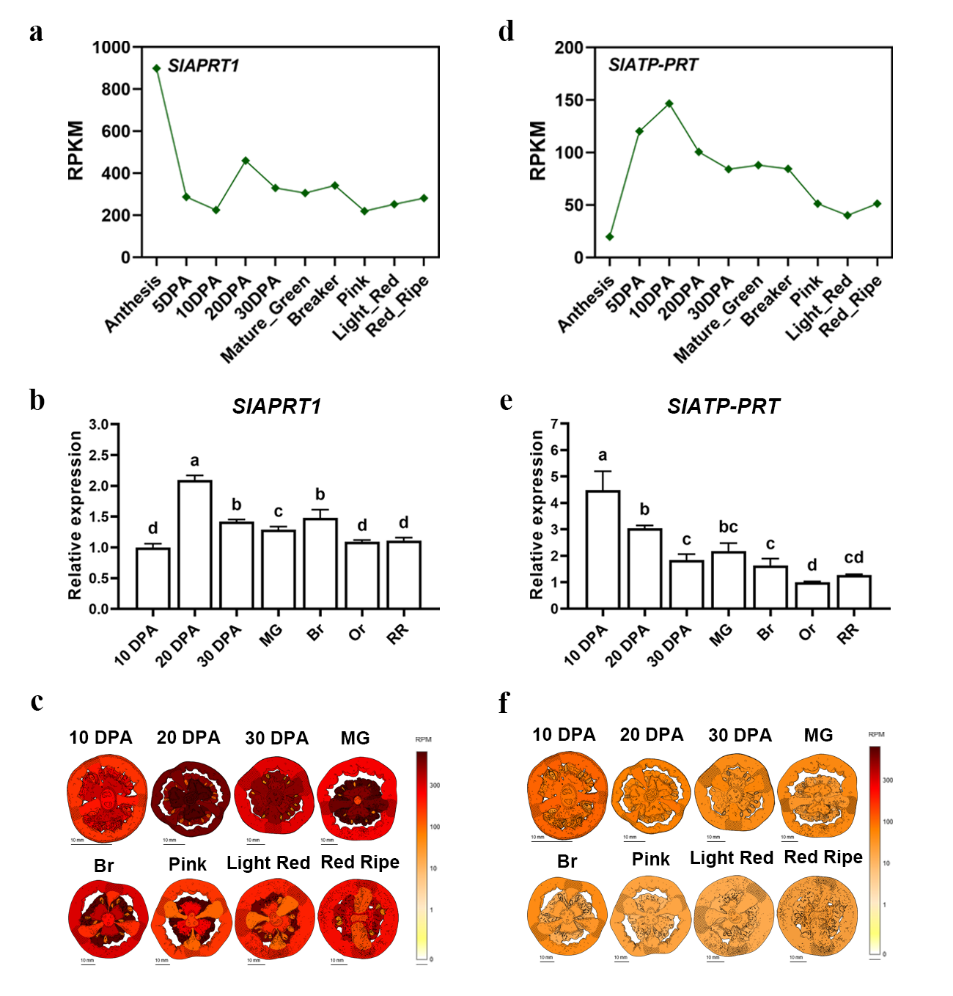


**Figure S2.** **Expression patterns of *SlAPRT1* and *SlATP-PRT* in tomato fruits from growth to ripening.**

**a**-**c**, Expression profiles of the *SlAPRT1* gene in the pericarp (**a-b**) and the equatorial plane (**c**) of tomato fruits.

**d**-**f**, Expression profiles of the *SlATP-PRT* gene in the pericarp (**d-e**) and the equatorial plane (**f**) of tomato fruits.

The data for (**a**) and (**d**) were sourced from Shinozaki et al. (2018), whereas the data for (**b**) and (**e**) were obtained from the RT-qPCR experiments conducted in this study. (**c**) and (**f**) were generated utilizing the Tomato Expression Atlas database. *SlACTIN* was used as a reference in (**b**) and (**e**). The relative transcription levels of the fruit at the developmental stage with the lowest expression level was set to 1.


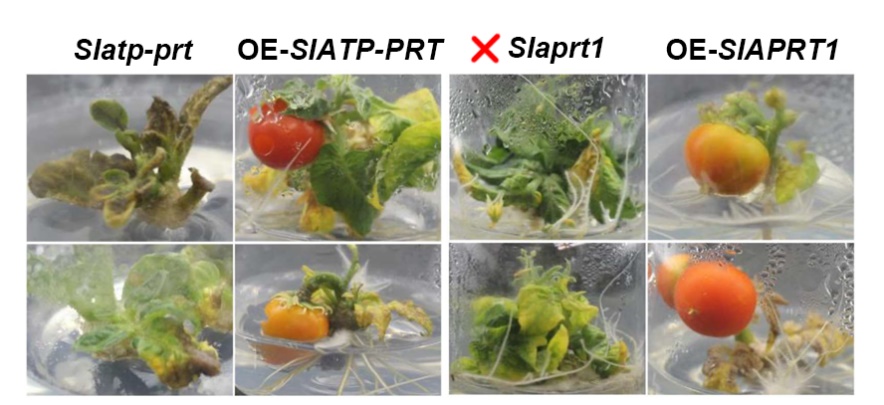


**Figure S3.** **Seedling phenotypes during tissue culture.**

X represents that no positive *SlAPRT1* knockout plants were obtained.


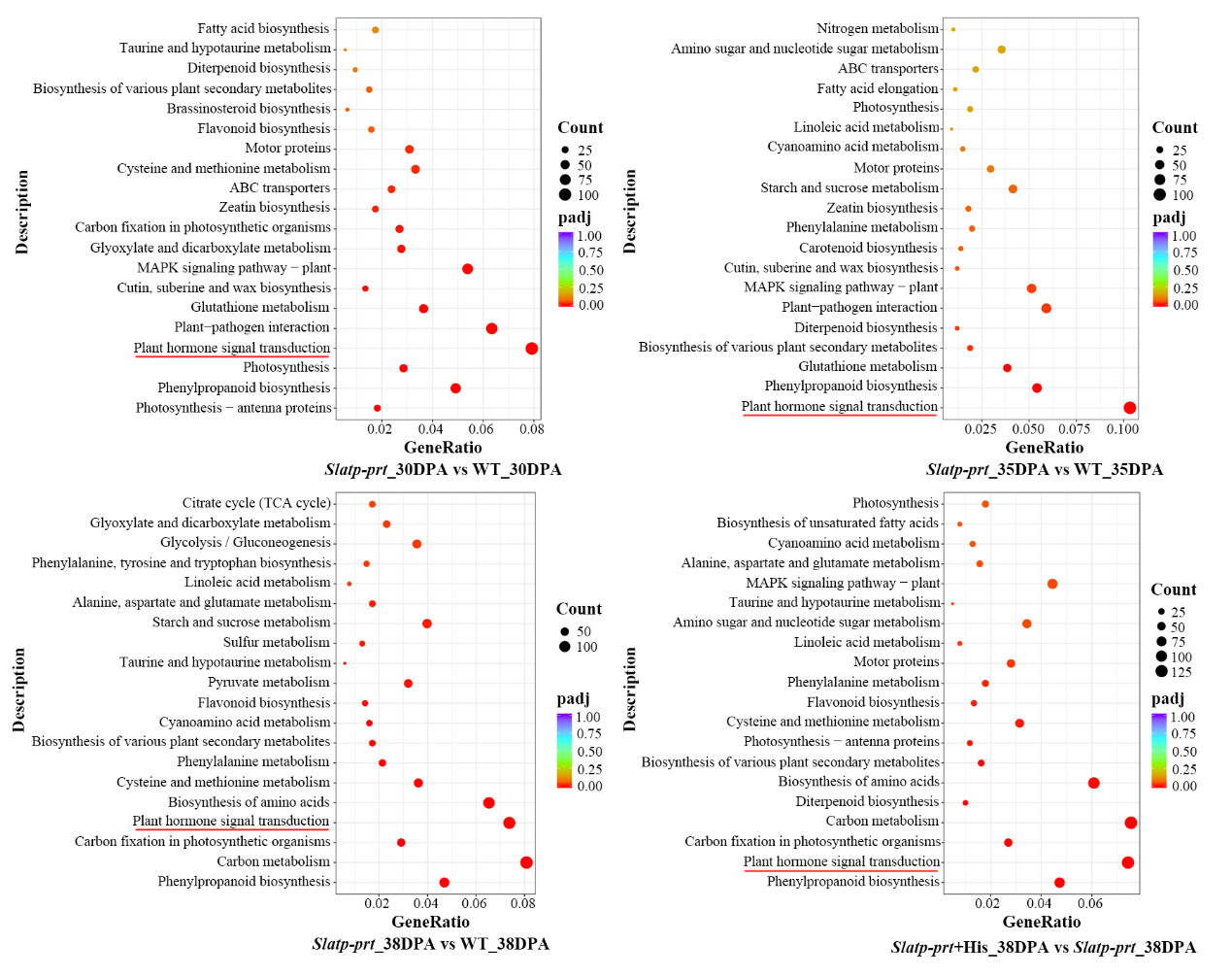


**Figure S4. KEGG pathway enrichment analysis of differentially expressed genes in *Slatp-prt* mutant fruits compared with wild-type fruits.**

Significantly enriched pathways (*padj* < 0.05) were identified for differentially expressed genes (DEGs) in *Slatp-prt* mutant fruits compared with wild-type fruits, as well as in mutant fruits treated with and without 100 μmol L⁻¹ His. The top 20 most enriched KEGG pathways are visualized in a scatter plot. X-axis: proportion of DEGs annotated to each pathway relative to total DEGs; y-axis: KEGG pathways; dot size: number of genes annotated to the pathway; color intensity: enrichment significance (red > purple).


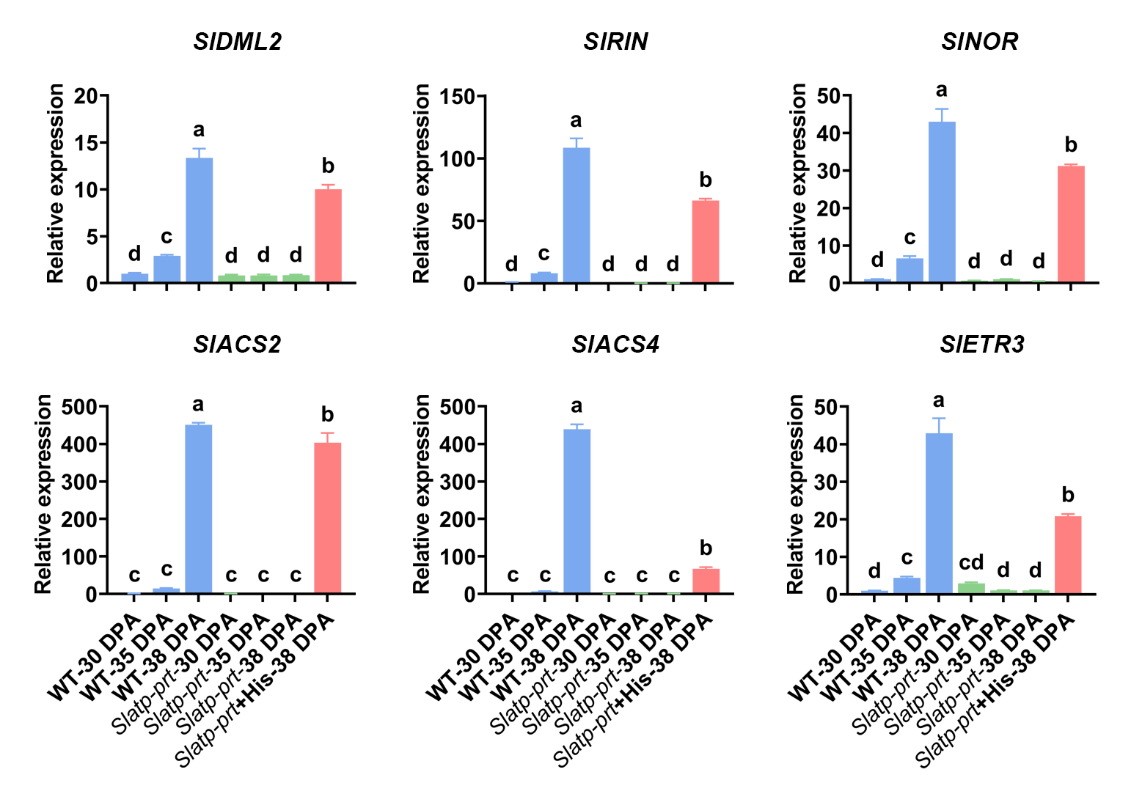


**Figure S5.** **The** **relative expression levels of key ripening-related genes in *Slatp-prt* mutant fruits compared with wild-type fruits.**

The relative expression levels of *SlRIN*, *SlNOR*, *SlACS2*, *SlACS4*, *SlDML2*, and *SlETR3* in tomato fruits at various developmental stages were analyzed by RT-qPCR in wild-type plants, the *Slatp-prt* mutant, and the *Slatp-prt* mutant irrigated with 100 μmol L^-1^ His. The accession numbers of these genes can be found in Table S3. The error bars represent ±SD for three biological replicates. Different letters indicate significant differences as determined by multiple comparisons with Duncan's test (*P* < 0.05). *SlACTIN* was used as a reference, and the relative transcription levels in the 30 DPA wild-type fruits were set to 1.


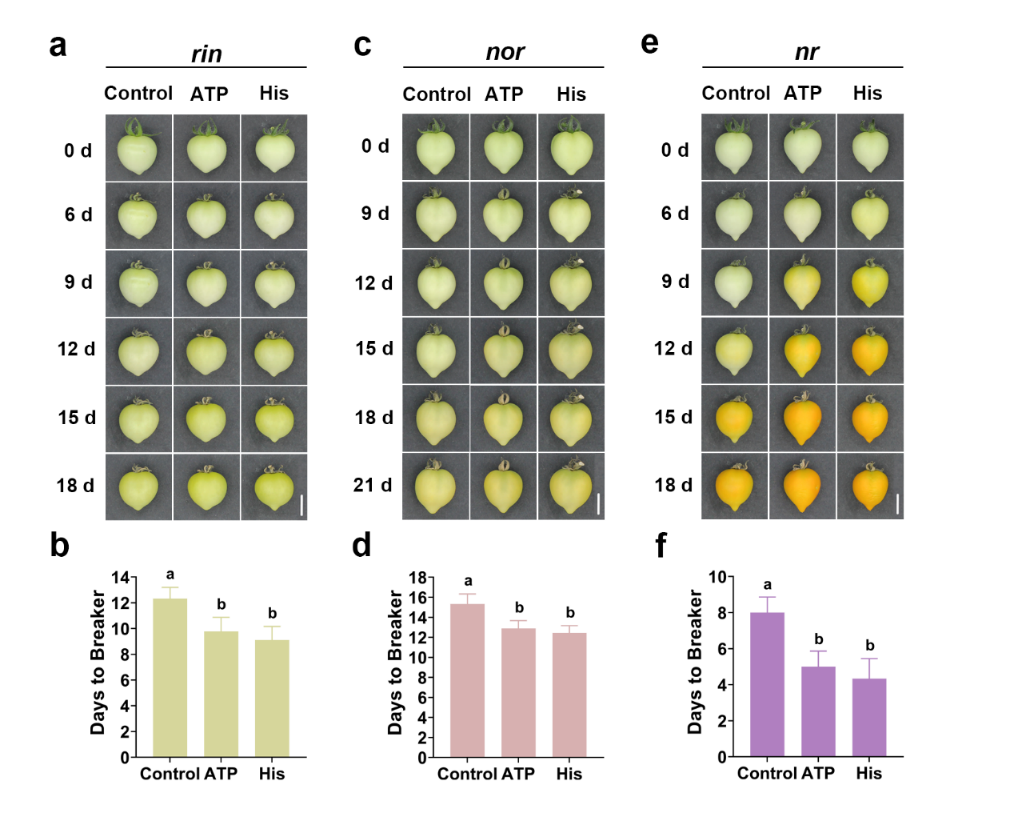


**Figure S6. Exogenous ATP and His induced ripening transition in harvested immature spontaneous tomato mutants.**

**a**-**b**, The ripening process from 0 to 18 days (**a**) and the Br stage (**b**) after the harvested *rin* mutant fruits were treated with 100 μmol L^-1^ ATP or His injection.

**c**-**d**, The ripening process from 0 to 21 days (**c**) and the Br stage (**d**) after the harvested *nor* mutant fruits were treated with 100 μmol L^-1^ ATP or His injection.

**e**-**f**, The ripening process from 0 to 18 days (**e**) and the Br stage (**f**) after the harvested *nr* mutant fruits were treated with 100 μmol L^-1^ ATP or His injection.

*Rin*, *nor*, and *nr* mutant fruits were harvested at 30 DPA. Bar = 1 cm, shown in the lower right corner of (**a**), (**c**), (**e**). The error bars represent ±SD for three biological replicates, and different letters indicate significant differences as determined by multiple comparisons with Duncan's test (*P* < 0.05).


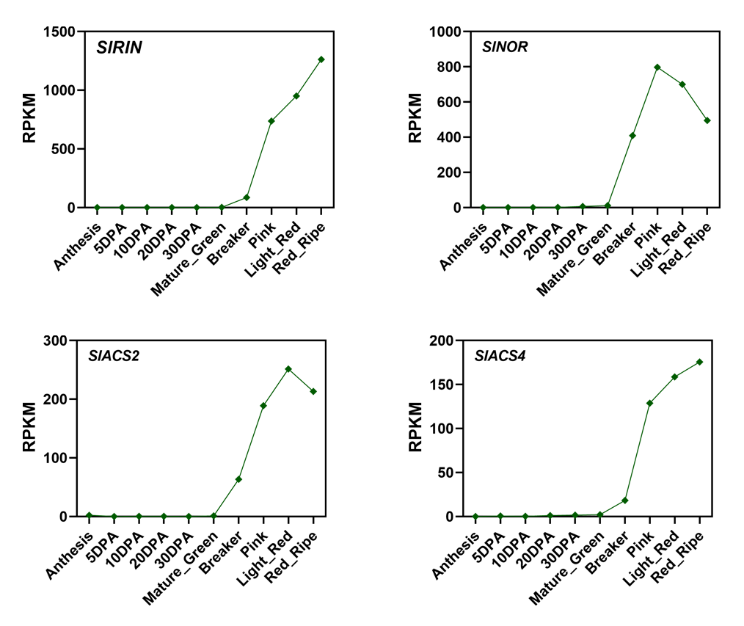


**Figure S7. Expression patterns of *SlRIN*, *SlNOR*, *SlACS2*, and *SlACS4* in tomato pericarp.**

The data were sourced from Shinozaki et al. (2018).
