# Supplementary material for: Regulation of the growth-to-ripening transition in tomato fruits by energy charge involving SlATP-PRT and SlAPRT1
Source: Mol Hortic. 2026 Jul 2;6:48. doi: 10.1186/s43897-026-00234-x (PMC13326308; doi:10.1186/s43897-026-00234-x)
Supplement: Supplementary file 3 — Additional file 3: Document S1. Supplementary methods in this study. [file 43897_2026_234_MOESM3_ESM.docx]

**The identification of transgenic plants**

After the transgenic seedlings grew, the leaves were harvested, and DNA was extracted using the Hi-DNAsecure Plant Kit (TIANGEN, DP350). The sequence encoding the *Hyg^R^* gene in T-DNA was amplified by Hyg-F and Hyg-R primers (Table S2) to detect whether the T0-generation transgenic seedlings were positive. Primers casYZ-F and casYZ-R (Table S2) were used to amplify the gene sequences near the *SlATP-PRT* target. The sequencing results were decoded using DSDecodeM (http://skl.scau.edu.cn/dsdecode/). Due to growth stagnation or decay observed in most T0-generation *SlATP-PRT* gene knockout seedlings, the seedlings were irrigated three times with 100 mL exogenous His solution (100 μmol L^-1^) after transplantation in the soil to obtain viable mutants.

**Determination of ATP, ADP, AMP, Ado, Ade, and free His contents**

The extraction of ATP, ADP, AMP, Ado, and Ade was conducted according to the previously established method with minor modifications (Zhou et al., 2022). A 0.2 mg sample of ground fruit was mixed with 1 mL of pre-cooled 0.4 moL L^-1^ perchloric acid, followed by ultrasonic disruption in an ice water bath. The resulting mixture was centrifuged at 12,000 ×*g* for 10 min at 4 ℃. Next, 100 μL of the supernatant solution was taken and quickly combined with 100 μL of 0.4 moL L^-1^ potassium hydroxide, and then centrifuged at 12,000 ×*g* for 10 min at 4 ℃. The supernatant obtained was analyzed using a Waters 2695 high-performance liquid chromatography equipped with an ultraviolet detector and a Sepax BR-C18 (4.6 × 250 mm, 5 μm) column at the detection platform of Beijing Biotech Pack Scientific Co., Ltd. (Beijing, China). The mobile phase consisted of 35 mmol L^-1^ sodium dihydrogen phosphate. Isocratic elution was performed for 50 min at a flow rate of 0.9 mL min^-1^. ATP, ADP, AMP, Ado, and Ade were identified and quantified based on the retention time and external standard curves. Subsequently, the fruit energy charge was determined using the formula (ATP + 1/2 ADP)/(ATP + ADP + AMP). Three distinct biological replicates of each experimental group were included.

The extraction method for free His is as follows: 50 mg of ground fruit sample was taken and mixed with 1 mL of 75% methanol, followed by vortexing for 1 h. The mixture was centrifuged at 12000 ×*g* at 4℃ for 5 min, and then 95 μL of supernatant was collected. Subsequently, 5 μL of phenylalanine internal standard solution (100 ng mL^-1^) was added to the supernatant, followed by thorough mixing. The prepared solution was analyzed using a Waters ACQUITY ultra-high performance liquid chromatography I-Class system coupled with a Waters XEVO TQ-S micro triple quadrupole mass spectrometer at the detection platform of Beijing Biotech Pack Scientific Co., Ltd. (Beijing, China). The quantification of His was based on comparing the response signal with the internal standard. Three distinct biological replicates of each experimental group were included.

**Treatment of harvested immature fruits with His, ethylene, and 1-MCP**

His, ethylene, and the combination of His and ethylene or 1-MCP were applied to harvested tomato fruits at the immature stage as follows: In the ethylene treatment group, approximately 200 μL of MES buffer (10 mmol L^-1^ pH 5.5) was injected into the upper columella of each fruit using a 1 mL sterile syringe. The fruit was then placed in a closed plastic box and fumigated with 50 ppm ethylene gas for 4 h. In the His treatment group, about 200 μL of MES buffer containing 100 μmol L^-1^ His was injected into the upper columella of each fruit, and then placed in a closed plastic box for 4 h. For the His+ethylene treatment group, about 200 μL of MES buffer containing 100 μmol L^-1^ His was injected into the upper columella of each fruit. The fruit was then placed in a closed plastic box and fumigated with 50 ppm ethylene gas for 4h. In the His+1-MCP treatment group, about 200 μL of MES buffer containing 100 μmol L^-1^ His was injected into the upper columella of each fruit. The fruit was then placed in a closed plastic box and treated with 20 ppm 1-MCP for 12 h; In the negative control group, approximately 200 μL of MES buffer was injected into the upper columella of each fruit, and then placed in a closed plastic box for 12 h. There were three biological replicates per group, totaling 9 fruits, all of which shared similar shapes, sizes, and colors. The plastic basket was covered with plastic wrap for moisturization and placed in a constant light greenhouse (16 h light/day). The injection was repeated three days later, and then the ripening phenotype was observed and recorded.
